# Prevalence, distribution and antimicrobial resistance profiles in poultry meat samples from India: a systematic review

**DOI:** 10.3389/fvets.2025.1672628

**Published:** 2025-10-31

**Authors:** V. Pooja Sajish, Nazim Uzzaman, Niroshini Aramvalarthan, Muhammad Asaduzzaman

**Affiliations:** ^1^Usher Institute, University of Edinburgh, Edinburgh, United Kingdom; ^2^Department of Community Medicine and Global Health, Institute of Health and Society, Faculty of Medicine, University of Oslo, Oslo, Norway

**Keywords:** antimicrobial resistance, chicken meat, one health, India, *Escherichia coli*, Salmonella, multidrug resistance

## Abstract

**Introduction:**

Chicken meat is a widely consumed source of protein in India but increasing reports of bacterial contamination and antimicrobial resistance (AMR) raise significant public health concerns. This systematic review aims to assess the prevalence of key bacterial pathogens in chicken meat across India and their resistance profiles.

**Methods:**

A comprehensive literature search was carried out across PubMed, Web of Science, and Scopus databases for studies published up to August 2024. Additionally, gray literature was retrieved using Google Scholar. Studies that identified bacterial isolates from chicken meat samples in India and reported antimicrobial susceptibility results were selected for inclusion. Data were extracted on bacterial species, sample location, antibiotics tested, and resistance rates using Microsoft Excel. A heatmap and summary tables were generated to visualize resistance trends.

**Results:**

A total of 32 studies were included in this review, with *Escherichia coli* and *Salmonella* spp. emerging as the most frequently detected pathogens. High resistance rates were observed to ampicillin, tetracycline, ciprofloxacin, and streptomycin. The overall multidrug resistance (MDR) rate exceeded 60% for several species. Resistance genes such as *bla*, *tet*, and *sul* families were frequently reported, along with virulence genes like *invA* and *icaA*.

**Conclusion:**

The widespread presence of multidrug-resistant bacteria in Indian chicken meat underscores the urgent need for robust surveillance, regulatory action on antibiotic use in poultry, and adoption of a One Health approach to mitigate AMR transmission.

## 1 Introduction

Ever since their discovery in the 20th century, antibiotics have revolutionized human and animal health by enabling the effective treatment of infectious diseases. Today, industrial agriculture heavily depends on antimicrobials—including antibiotics, antifungals, and antiprotozoals—to manage and prevent diseases, enhance animal welfare, and boost productivity (1). However, this heavy dependence, and at times over-dependence, has ironically become a global threat to both animal and human health due to the rise of antimicrobial resistance (AMR) (2). AMR refers to the capacity of microorganisms to withstand antimicrobials that they were once susceptible to, thereby diminishing the effectiveness of treatments (3). Following the continuous exposure and usage of antibiotics, numerous microorganisms evolve in an attempt to survive or dodge current and new antimicrobials, resulting in short to long-term resistance. While the evolution of AMR is a natural process, the excessive and improper use of antimicrobials can significantly accelerate it, leading to a swift proliferation of resistant bacteria in the environment (4). A recent global disease burden study reported that AMR caused 1.27 million direct deaths in 2019 and was a contributing factor in 4.95 million deaths globally (5). In Southeast Asia alone, it is estimated that more than 97,000 people died directly due to AMR in 2019 (5). Projections also indicate that AMR will lead to 4.7 million deaths in Asia by 2025 (6).

AMR is a multifaceted One Health (OH) issue which has enormous impact on not only public health but also animal health and environment due to their profound role and impacts in the whole resistance process. Antibiotics are administered to food-producing livestock for several purposes, including non-therapeutic applications, managing diseases within a herd or flock, and treating infectious diseases (7). In some countries, around 80% of the total consumption of medically important antibiotics occurs in the animal sector, primarily to promote growth in healthy animals (8). In fact, the use of antimicrobials in food-producing animals has expanded in the previous decades due to a rise in global demand for animal protein (2), as well as for cultural shifts (9). It is projected that such use is expected to increase by 8% to 107,472 tons by 2030 (10). The presence of antibiotic residues in food is primarily derived from poultry (chicken meat and eggs) (11–13), cattle (beef, milk, and meat) (14–17), and pigs (pork) (18, 19). Various factors contribute to the presence of these residues in food. Sharma et al. (20) outlined a model indicating that both direct and indirect interactions between humans and animals can lead to antibiotic residue transmission. Direct transmission happens when antibiotics administered to animals or applied to crops remain in the food after slaughter or harvest (21). Conversely, indirect transmission occurs when residues from manure (containing antibiotics from animals) or human waste (containing antibiotic residues) accumulate in water and soil, eventually contaminating food, particularly vegetables, through irrigation or contact with animal feces (22). While the latter is less common, numerous studies have detected considerable antibiotic residues in soil and manure, suggesting these residues can end up in plant-based foods as well (23–25). Such transmissions are significant contributors in OH-AMR where large human and animal population exist with increasing demand for animal protein, particularly, in South Asia. It is not surprising that several countries in the region, including Bangladesh, India, Indonesia, Nepal, Sri Lanka, and Thailand, have reported a concerning rise in AMR (26). It is alarming that AMR could lead to 2 million deaths only in India by 2050 (27).

In line with global trends, poultry in India has emerged as the leading source of meat, now representing 65 percent of total meat consumption, a significant increase from 23 percent two decades ago (28). This growth has outpaced other competitors such as beef, and buffalo meat (29). Additionally, poultry consumption is projected to increase by 577% between 2000 and 2030 (30). Currently, India is world’s third largest egg producer falling behind China and USA, and the world’s fifth largest poultry producer, following the USA, Brazil, China and the European Union (31). Specific regions in India have emerged as key hubs for the poultry industry, with Tamil Nadu, Andhra Pradesh, and Telangana leading the country, closely followed by Maharashtra, Punjab, and West Bengal (31). Several factors contribute to India’s poultry dominance: high mutton prices, religious restrictions on beef and pork, and limited fish availability outside coastal areas have all driven poultry to become the preferred and consumed choice for many (28). To meet the rising demand, there is a push in using antibiotics in food animals, and as a result, antibiotic consumption in food for animal production in India is expected to increase by 312% by 2030, which makes India the fourth largest user of antibiotics in animals (32). In this way, India is particularly vulnerable to AMR context, liked with high antibiotic consumption in animal food value chain.

Given the widespread emergence of OH-AMR in India, it is essential to understand the prevalence and patterns of antibiotic resistance in food animal and animal products, especially in poultry meat. A comprehensive evidence synthesis of drug-resistant bacteria in poultry meat in India would provide a national overview of OH-AMR burden. Therefore, a systematic review was undertaken to identify the total incidence of bacterial pathogens in chicken meat and meat products from abattoirs and retail markets in India, as well as their antimicrobial resistance profiles. Although previous reviews have addressed antimicrobial resistance (AMR) in poultry (33–35), this study represents, to our knowledge, the first systematic review dedicated exclusively to chicken meat in India from various sources such as retail shops, local markets, slaughterhouses, and repositories. This review maps the geographic distribution of bacterial pathogens and antimicrobial resistance patterns across India, with a notable concentration of data from the southern states. It integrates findings from both peer-reviewed studies and gray literature, offering a broader and more inclusive evidence base. By extending the review timeline to include publications up to 2024, it provides an updated and comprehensive synthesis of bacterial prevalence, multidrug resistance (MDR) indices, and associated resistance and virulence genes. Additionally, the review contextualizes regional disparities within the framework of India’s National Action Plan on AMR, linking observed trends to existing national policies.

## 2 Methods

### 2.1 Study protocol

**Figure 1:**
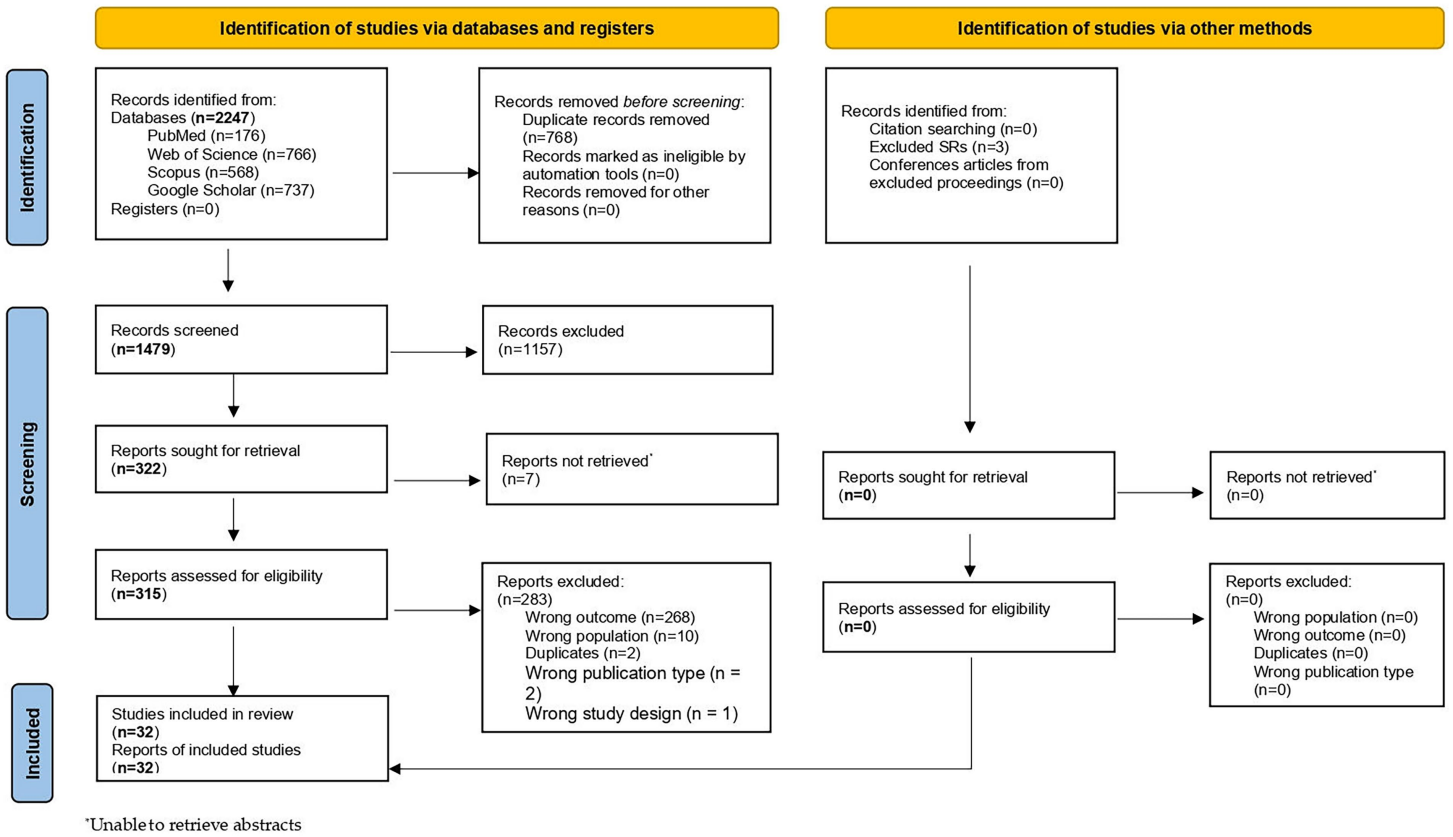
PRISMA flowchart^34^.

The research protocol was developed *a priori* and registered ahead of data collection on the Open Science Framework.^1^ The identification of records, the screening of titles and abstracts, and the assessment of full texts for final inclusion were conducted in accordance with the PRISMA (Preferred Reporting Items for Systematic Reviews and Meta-Analyses) checklist. Furthermore, this systematic review was reported using the PRISMA guidelines (36) and the PRISMA for Abstracts checklist (37). The PRISMA checklists for the abstracts are provided in Supplementary Table S1. Additionally, a PRISMA flow diagram (Figure 1) was used to record each step, ensuring a transparent and reproducible methodology.

### 2.2 Data sources and search strategies

The primary reviewer (PS) conducted a systematic search of PubMed, Web of Science, and Scopus to identify relevant primary studies. The search was carried out without any (e.g., language or date) restrictions from inception to August 12th, 2024. The search strategies included important key words and indexing terms: Poultry (MeSH), “Poultry products” (MeSH), bacteria*, “antimicrobial resistance,” “antibacterial resistance,” “antimicrobial susceptibility,” “India” (MeSH), “Bharat,” and “Hindustan.” Boolean logical connectors, namely “AND” and “OR” were utilized in the search to combine and refine the keywords appropriately. Truncation was also employed to capture variations of the search terms, expanding the scope of identification for relevant records. The basic search strategy employed in PubMed was modified and customized for use in the other databases. The specific search strategies for each database are provided in Supplementary Table S2. To ensure comprehensiveness, we searched for gray literature on Google scholar. Furthermore, hand-searching of the reference lists of included studies were also conducted to identify any additional relevant studies.

### 2.3 Screening and eligibility of studies

Using the online systematic review software Rayyan (38), PS removed all identified duplicates, following which PS and NA independently conducted title and abstract screening. The titles and abstract screening were facilitated by pre-defined inclusion and exclusion criteria established based on the PEO (population, exposure, outcome) format. In terms of population (P), the review considered studies that focused on chicken meat as the subject of investigation. The exposure (E) of interest involves bacterial pathogens, examining studies that explore the presence of pathogens in relation to chicken meat. The outcome (O) of interest is the prevalence of antimicrobial resistant bacterial pathogens in chicken meat, as well as the antimicrobial resistance patterns observed. The geographical setting (S) for inclusion will be limited to studies conducted within India. After title and abstract screening, PS independently assessed eligibility of the articles reading the full texts and NA checked all the excluded articles. Discrepancies at both stages were resolved by a third reviewer (MA).

### 2.4 Data extraction

Data extraction was independently done by PS and checked by NA. After piloting with a small study sample, an iterative method was employed to create a standardized table for extracting pertinent information. A summary of information taken from each study includes the following: (i) study design (ii) location (iii) setting (iv) type of sample (v) sample size (vi) study duration (vii) study outcomes (viii) funding and conflicts of interest. To resolve any disagreements, PS, NA, and MA met to reach a consensus.

### 2.5 Outcome measurements

The main focus of this study is to determine the prevalence of clinically significant bacterial isolates in meat and meat products collected from abattoirs and retail establishments across India. This primary outcome measure aims to identify the presence and abundance of drug-resistant bacterial strains that could potentially pose a risk to public health. Additionally, the study also examined the antimicrobial resistance status of the identified bacterial isolates. Specifically, the resistance levels of these bacteria against selected antimicrobials from different categories, including ceftriaxone, gentamicin, ciprofloxacin, and ampicillin, were assessed. Multidrug resistance (MDR) was defined as resistance of an isolate to three or more antimicrobial classes (39). The proportion of MDR isolates was calculated as:


$$\mathrm{MDR}\%=\frac{\begin{array}{l} \text{Number of isolates resistant to atleast}\;1\;\\ \text{agent in}\geq 3\;\text{antimicrobial classes} \end{array}}{\text{Total number of isolates}}*100$$

The Multiple Antibiotic Resistance (MAR) index for each isolate was calculated as:


MARindex=a/b

where *a* = number of antibiotics to which the isolate was resistant, and *b* = total number of antibiotics tested for that isolate. This secondary outcome measure aims to shed light on the resistance patterns of the bacterial strains found in the meat samples, which is crucial for understanding the potential threats they pose in terms of antibiotic treatment efficacy and the development of antimicrobial resistance (Table 1).

**Table 1:** Summary of antimicrobial resistance pattern among bacterial isolates in the included studies.

Bacterial isolate	# of studies	Most tested antibiotics	Sample size range	Range of resistance (%)
*Aeromonas*	2	Gentamicin, Chloramphenicol, Ciprofloxacin, Nalidixic acid, Ampicillin, Cephotaxime, Kanamycin, Cefuroxime	15–104	0–100
*Campylobacter*	1	Doxycycline, Erythromycin, Ciprofloxacin, Nalidixic acid, Tetracycline, Ampicillin, Amoxyclav, Kanamycin	275	26.3–68.4
*Escherichia coli*	14	Norfloxacin, Colistin, Erythromycin, Gentamicin, Penicillin-G, Enrofloxacin, Streptomycin, Ceftriaxone, Amoxycillin, Chloramphenicol, Nalidixic acid, Tetracycline, Ampicillin, Trimethorpim, Methicillin, Vancimycin, Cotrimoxazole, Nitrofurantoin, Cefazolin, Cephotaxime, Amoxyclav, Oxytetracycline, Imipenem, Cefpodoxime, Ceftazidime, Amikacin, Fosfomycin, Aztreonam, Cefoperazone	5–228	0–100
*Enterobacter*	1	Colistin, Gentamicin, Ciprofloxacin, Trimethoprim, Imipenem, Ceftazidime, Amikacin, Levofloxacin, Meropenem, Aztreonam, Cefoperazone, Cefipime, Doripenem, Tigecycline, Minocycline	32	0
*Klebsiella*	3	Bacitracin, Rifampicin, Colistin, Doxycycline, Gentamicin, Penicillin-G, Streptomycin, Ciprofloxacin, Ampicillin, Cephotaxime	5–32	0–100
*Listeria*	1	Norfloxacin, Bacitracin, Rifampicin, Colistin, Doxycycline, Erythromycin, Gentamicin, Penicillin-G, Sulphadiazine, Enrofloxacin, Streptomycin, Ceftriaxone, Amoxycillin, Chloramphenicol	100	11.6–90.7
*Micrococcus*	1	Bacitracin, Rifampicin, Erythromycin, Penicillin-G, Streptomycin, Chloramphenicol, Ciprofloxacin, Ampicillin, Vancomycin, Cephotaxime	5	0–100
*Proteus*	2	Streptomycin, Chloramphenicol, Ciprofloxacin, Ampicillin, Vancomycin, Cotrimoxazole, Beta-lactam, Cephotaxime	5–195	0–100
*Pseudomonas*	1	Colistin, Gentamicin, Trimethoprim, Imipenem, Ceftazidime, Amikacin, Meropenem, Tazobactam, Cefoperazone, Cefipime, Tigecycline, Minocycline, Clavulanic acid	32	0–100
*Salmonella*	9	Rifampicin, Colistin, Erythromycin, Gentamicin, Enrofloxacin, Streptomycin, Amoxycillin, Chloramphenicol, Ciprofloxacin, Nalidixic acid, Tetracycline, Ampicillin, Vancomycin, Cotrimoxazole, Nitrofurantoin, Cephotaxime, Oxytetracycline, Cephalexin, Furazolidone, Amikacin, Ceftriaxone	5–578	0–100
*Shigella*	1	Bacitracin, Rifampicin, Erythromycin, Penicillin-G, Streptomycin, Chloramphenicol, Ciprofloxacin, Ampicillin, Vancomycin, Cephotaxime	5	50–100
*Staphylococcus aureus*	9	Rifampicin, Doxycycline, Erythromycin, Gentamicin, Penicillin-G, Streptomycin, Chloramphenicol, Ciprofloxacin, Tetracycline, Ampicillin, Trimethoprim, Methicillin, Vancomycin, Polymixin-B, Novobiocin, Cephotaxime, Amoxyclav, Cefoxitin, Linezolid, Ofloxacin, Cloxacillin, Kanamycin	5–147	0–100

Here, ‘most tested antibiotics’ denotes the antibiotics that were most frequently assessed across the included studies for that particular bacterial isolate. This table summarizes the number of studies reporting each bacterial species, the most commonly tested antibiotics, and the range of resistance rates observed. See Supplementary Table S4 for full details.

### 2.6 Data synthesis

The summary of findings is presented in the form of a narrative synthesis due to several reasons. First, initial scoping searches indicate substantial heterogeneity among study data, rendering a meta-analysis unsuitable and prone to yielding inaccurate and misleading outcomes. Second, variables like rates, locations, identified bacteria, and analytical methodologies might have undergone fluctuations over time. This could potentially obscure significant associations between microbial exposure changes and prevalence rates. Narrative synthesis refers to an approach in systematic reviews in which the results from the studies that have been included are primarily explained through text. This narrative synthesis was done according to the ‘Guidance on the Conduct of Narrative Synthesis in Systematic Reviews’ by Popay et al. (40).

## 3 Results

A comprehensive and systematic search yielded a total of 2,247 records. After eliminating 768 duplicates, 1,479 unique studies were subjected to title and abstract screening. This phase narrowed the selection down to 322 articles that were eligible for full-text review. Following the application of inclusion and exclusion criteria, 32 studies were finally included in this systematic review. The PRISMA flow diagram (Figure 1) illustrates the selection process, and a detailed summary of the characteristics of each study is presented in Supplementary Table S3.

**Figure 2:**
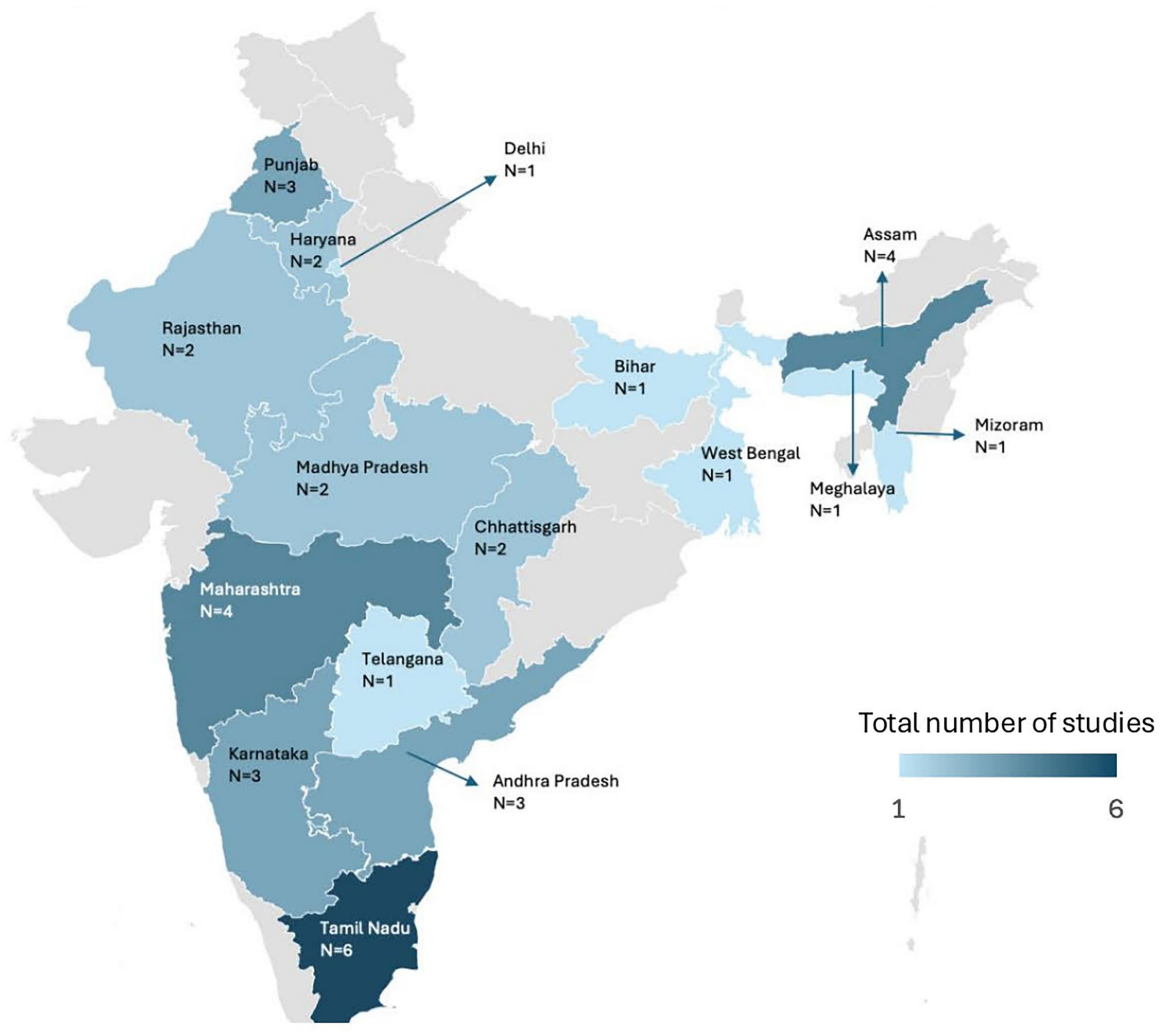
Geographical distribution of studies reporting bacterial pathogens in chicken meat across india. The total count of studies shown in the figure exceeds the number of included studies because three studies reported data from multiple states.

The included studies spanned several Indian states and union territories, with South India being the most represented region. Tamil Nadu contributed the highest number of studies (6 out of 32, 18.75%), followed by Assam and Maharashtra (4 each), and Andhra Pradesh, Karnataka, and Punjab (3 each). States such as Bihar, Meghalaya, Mizoram, Telangana, West Bengal, and the NCT of Delhi each had one study. Notably, several regions, including Goa, Gujarat, Kerala, Odisha, and parts of the northeastern and Himalayan zones, had no studies. Regional representation is visually depicted in Figure 2.

**Figure 3:**
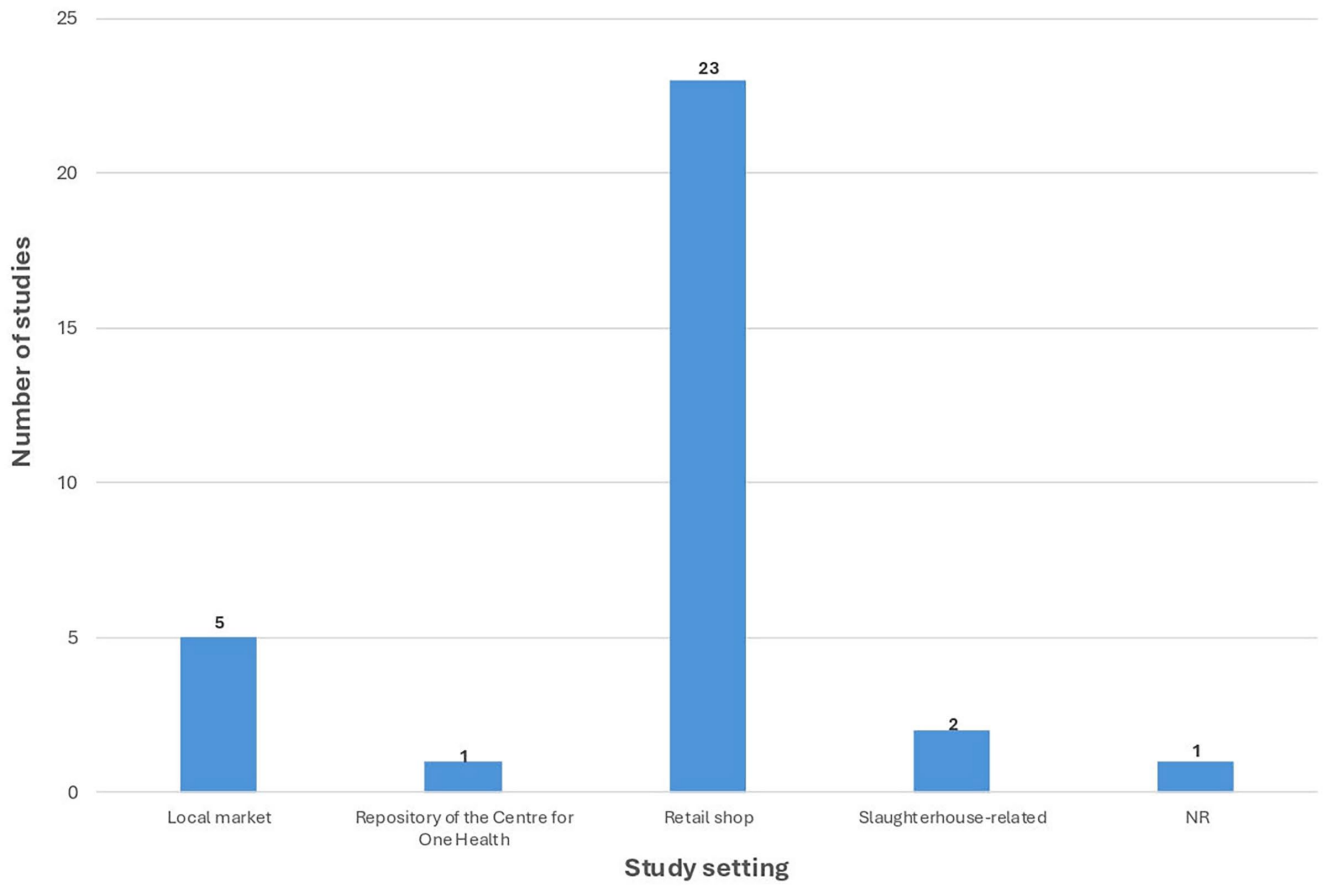
Distribution of study settings in included studies on bacterial pathogens in indian chicken meat. NR, Not reported.

Figure 3 illustrates the distribution of study settings among the included articles. The majority of studies (*n* = 23) collected samples from retail shops, followed by local markets (*n* = 5). Two studies were conducted in slaughterhouse-related settings; for reporting clarity, data from “Slaughterhouse” (*n* = 1) and “Slaughterhouse and Retail meat shop” (*n* = 1) were grouped under a single category. One study used samples from the Repository of the Centre for One Health, and one study did not report the study setting.

For clarity of reporting, the included studies were categorized based on the type of bacterial isolates, namely Gram-negative and Gram-positive. Furthermore, the studies were described according to their geographical distribution across different regions of India, as understanding regional patterns of AMR in isolates may provide insights into the historical development of resistance and help predict future regional spread and potential outbreaks.

### 3.1 Evidence of antibiotic-resistant gram-negative bacterial pathogens

**Figure 4:**
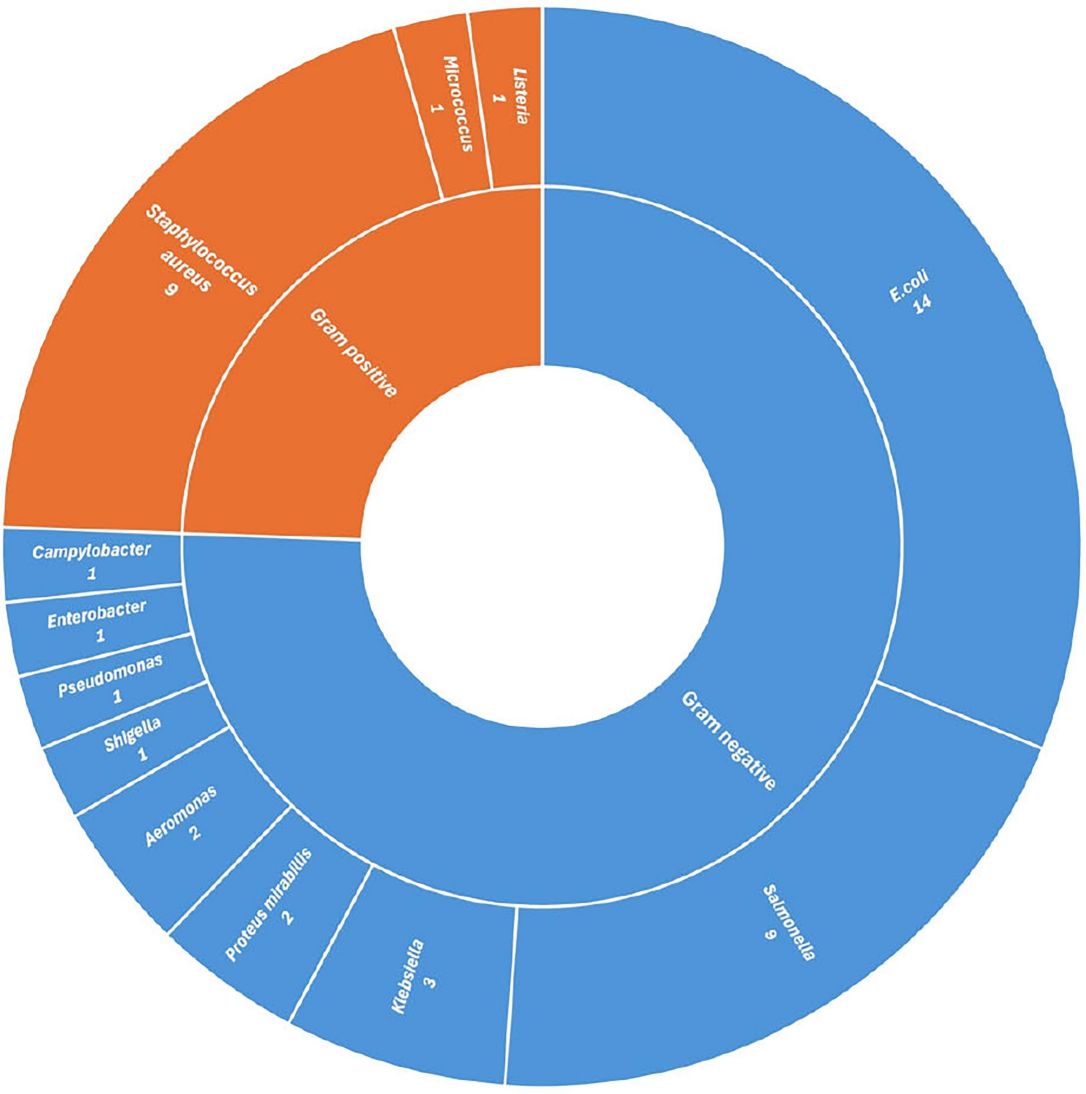
Distribution of studies reporting bacterial isolates from chicken meat in India, grouped by Gram-positive and Gram-negative classification.

***Escherichia coli (E.coli)***: Among the gram-negative bacterial isolates, *Escherichia coli* was the most prevalent and was identified in 14 studies (41–54) (43.75%) with three studies from Tamil Nadu (52–54) (see Figure 4). In Modak’s 2014 study, 4 out of 5 broiler meat samples from a local market tested positive for *E. coli*, with isolates exhibiting 100% resistance to commonly used antibiotics such as ampicillin, penicillin g, streptomycin, vancomycin, and several others (52). Similarly, Natarajan et al. identified *E. coli* in 14 out of 32 meat samples, including both retail and frozen sources. These isolates demonstrated 100% resistance to trimethoprim/sulfamethoxazole and 50% resistance to fluoroquinolones like gentamicin, levofloxacin, and ciprofloxacin, though they remained fully susceptible to several high-end antibiotics such as imipenem, meropenem, and colistin (53). Vasanthi’s et al. study further supports the concern, with 20 out of 30 meat swabs testing positive for *E. coli*. These strains showed high resistance to tetracycline (89%), methicillin (78%), penicillin (72%), and cefotaxime (61%). The calculated MAR (Multiple Antibiotic Resistance) index for these studies ranged from 0.25 to 1, with values ≥0.2 (54). Two studies conducted by Deka in Assam—one in 2008 and the other in 2022—provide valuable insights into the changing prevalence and AMR patterns of *E.coli* in retail chicken meat. In 2008, *E. coli* was isolated from 54 out of 100 meat samples (54% prevalence) (44), while in 2022, the prevalence had surged to 98 out of 110 samples (89%) (45). The resistance profiles also reflect concerning trends. In 2008, *E. coli* isolates showed 100% resistance to ampicillin, and notably high resistance to tetracycline (66.67%), nitrofurantoin (68.52%), and streptomycin (61.11%). Resistance to critically important antibiotics like ciprofloxacin and chloramphenicol remained moderate (22.22%) (44). However, in 2022, although resistance to ampicillin dropped slightly to 76%, resistance to tetracycline increased to 82% and nalidixic acid to 80%. Worryingly, resistance to third-generation cephalosporins like cefotaxime (10%) and newer agents like imipenem and amikacin remained at 0%, suggesting they are still effective. The MAR index remained alarmingly high (1.0 in 2008, 0.9 in 2022) (45).

In a 2018 study conducted by Kaushik in Bihar reported *E.coli* in 27% of 228 samples. The antimicrobial resistance AMR pattern revealed high resistance to several commonly used antibiotics. Notably, *E. coli* isolates exhibited resistance to cefuroxime (89.1%), penicillin (89.4%), ampicillin (80.43%), and vancomycin (74.1%), indicating extensive resistance to *β*-lactam and glycopeptide classes of antibiotics. Resistance to co-trimoxazole (73.1%) and cephalothin (60.8%) was also significant. However, resistance to higher-generation antibiotics remained comparatively low, with ceftriaxone at 28.2%, tetracycline at 17.4%, and gentamicin, amikacin, and ofloxacin all below 14%. ciprofloxacin had the lowest resistance rate (6.5%) (50).

Both Bhave et al. and Londhe et al. conducted studies in Maharashtra to assess *E.coli* contamination and AMR in chicken meat sourced from retail shops. Bhave studied 54 meat samples and found *E. coli* in 32 (59.3%) (41), while Londhe analyzed 106 samples with 45 positives (51). The proportion of resistant isolates was slightly higher in Bhave’s study (0.9) than in Londhe’s (0.86). Both studies reported universal resistance to nalidixic acid and high resistance to tetracycline (100% in Bhave, 86.66% in Londhe) (41, 51). ciprofloxacin resistance was similar (Bhave: 46.87%, Londhe: 51.11%). Bhave, however, reported 0% resistance to chloramphenicol, in contrast to Londhe’s 53.33%. Bhave detected multiple beta-lactamase genes (*blaTEM*, *blaCTX-M*, *blaOXA*). In contrast, Londhe identified the *tetA* gene, reflecting tetracycline resistance.

The study by Chakravarty et al. was conducted in Andhra Pradesh from chicken meat samples collected from a slaughterhouse. While the exact number of samples and prevalence rate were not reported (NR), antimicrobial susceptibility testing was performed on *E.coli* isolates. The isolates showed 100% resistance to both penicillin and ampicillin. Similarly, tetracycline resistance was also 100%. On the other hand, there was 0% resistance to gentamicin and co-trimoxazole. Moderate resistance (50%) was observed for ciprofloxacin, chloramphenicol, norfloxacin, nalidixic acid, and nitrofurantoin. The MAR index was 0.5 (42).

In a 2022 study by Jhandai conducted in Haryana, *E.coli* was isolated from 63 chicken meat samples collected from local markets. The isolates exhibited high resistance to penicillin (98.41%), amoxyclav (93.65%), cefotaxime (85.71%), cefpodoxime (93.65%), ceftazidime (87.3%), ceftriaxone (74.6%), and aztreonam (66.67%). Resistance to imipenem was observed in 90.48% of the isolates. High resistance was also reported to erythromycin (98.41%) and tetracycline (93.65%). Moderate resistance was found for amikacin and gentamicin (both 55.56%), streptomycin (69.84%), and chloramphenicol (46.03%). The overall MAR reached 0.98 (48).

The study by Debbarma et al. was conducted in Mizoram isolated from chicken meat samples collected from retail shops. The study examined four sets of meat samples collected from various urban and rural regions in Mizoram, totaling 180 samples, with sample sizes of 90, 30, 30, and 30, respectively. The *E. coli* prevalence rates ranged from 63.33 to 90%, with an average MAR index of 0.92. The isolates demonstrated consistently high resistance to beta-lactam antibiotics, including amoxicillin (84–85%), ampicillin (78–81%), and cefazolin (73–96%). High resistance was also observed to tetracycline (ranging from 31.5 to 88.8%) and cotrimoxazole (up to 85%). Fluoroquinolone resistance was comparatively lower, with ciprofloxacin resistance ranging from 19 to 40%, and norfloxacin from 7 to 15%. Resistance to gentamicin varied widely (10.5–55.5%), and resistance to amoxicillin-clavulanic acid was notably lower in some subsets (5.2–26.9%) (43).

Hussain et al. conducted a study across four southern Indian states—Karnataka, Telangana, Andhra Pradesh, and Maharashtra—examining the prevalence and antimicrobial resistance patterns of *E. coli* isolated from chicken meat sold in retail shops. In broiler chicken samples (*n* = 32), a total of 54 *E. coli* isolates were recovered, indicating multiple isolates per sample and a high contamination rate. These isolates exhibited significant resistance to commonly used antibiotics, including ciprofloxacin (96%), tetracycline (93%), co-trimoxazole (61%), and gentamicin (43%), while lower resistance was noted to chloramphenicol (9%) and fosfomycin (6%). Genetic analysis showed a high prevalence of extended-spectrum beta-lactamase (ESBL) genes, with *blaCTX-M-15* detected in 84% of isolates, *blaTEM* in 68%, and *blaSHV* in 32%. In contrast, *E. coli* was found in all 13 free-range chicken samples (100% prevalence), but with a distinct AMR profile. These isolates demonstrated high resistance to tetracycline (92%) but showed much lower resistance to ciprofloxacin (15%), co-trimoxazole (8%), and gentamicin (8%), and no resistance to chloramphenicol or fosfomycin. Despite the lower phenotypic resistance in free-range chicken, molecular testing revealed that all isolates harbored *ESBL* genes—*blaCTX-M-15* and *blaTEM* (100%), and *blaSHV* (50%) (46).

In a 2020 study by Kalwaniya conducted in Rajasthan, *E.coli* was isolated from 17 chicken meat samples obtained from retail shops. The isolates demonstrated complete resistance to erythromycin (100%) and high resistance to nalidixic acid (64.7%) and oxytetracycline (64.7%). Moderate resistance was observed against ampicillin (52.94%) and amoxyclav (52.94%), while lower resistance rates were reported for enrofloxacin (35.29%), chloramphenicol (29.41%), cotrimoxazole (29.41%), and ceftriaxone (11.76%). No resistance was detected to gentamicin (0%). The overall MAR was 0.9 (49).

Finally, Jana and Mondal investigated antimicrobial resistance patterns in *E.coli* isolated from chicken meat samples (*n* = 83) collected at retail shops in West Bengal. Out of these, 33 *E.coli* isolates were identified. The study revealed a concerning level of resistance to multiple antibiotics. Notably, 100% of the isolates were resistant to novobiocin, while high resistance rates were also observed for cefixime, sulphafurazole, and vancomycin (each at 92.31%). Substantial resistance was found against oxytetracycline (84.62%), erythromycin and methicillin (61.54% each). In contrast, no resistance was observed to amikacin or chloramphenicol, and only low levels of resistance were reported for kanamycin (7.69%), gentamicin (15.38%), and ciprofloxacin (15.38%) (47).

***Salmonella:*** *Salmonella* species (spp.) was reported in 9 studies (44, 52, 53, 55–60) (28.12%), and five studies of which were conducted in Tamil Nadu (52, 53, 56, 57, 61). These studies conducted in Tamil Nadu reported the isolation of *Salmonella* spp. from chicken meat samples collected from various sources such as retail shops and local markets. The prevalence of *Salmonella* isolates varied widely, ranging from 4 to 110. Indrajith et al. (61) observed the highest prevalence, with 110 isolates from 40 meat samples, followed by Maripandi et al., who reported 92 isolates in 578 meat samples (57). In contrast, Natarajan et al. (53) reported the lowest salmonella isolates of 4 among 32 samples. Notably, antimicrobial resistance patterns indicated a high level of resistance to commonly used antibiotics such as ampicillin, vancomycin, and rifampicin across most studies. Complete resistance to ciprofloxacin was observed in one study (52), while others reported full susceptibility, reflecting regional variation. Resistance to other agents like streptomycin, erythromycin, and kanamycin was also frequently reported. The multidrug resistance (MDR) index ranged from 0.35 to 1.0. The studies conducted in other parts of India highlight the widespread prevalence of *Salmonella* in chicken meat and the alarming levels of AMR associated with these isolates. Prevalence rates varied significantly, ranging from 8% in Assam (44) to as high as 46.7% in Himachal Pradesh (55), with sample sizes spanning from 30 to 240. The antimicrobial resistance profiles indicate a troubling trend of MDR among *Salmonella* strains. For instance, Deka et al. reported 100% resistance to ampicillin and over 87% resistance to tetracycline and cephalexin (44), while Mishra and Shukla found >90% resistance to multiple tetracycline antibiotics and amoxycillin (58). Naik et al. observed complete resistance to erythromycin, and Rajashekhara et al. noted total resistance to key antibiotics such as ciprofloxacin, cefotaxime, and ceftriaxone. MAR indices ranged from 0.1176 to 0.3529, with values ≥0.2 (59).

***Klebsiella:*** *Klebsiella*species were isolated in three separate studies conducted in Tamil Nadu (52–54), each highlighting varying degrees of AMR in chicken meat samples. The findings present a mixed picture, ranging from extreme resistance to complete susceptibility. In the earliest study by Modak et al., *Klebsiella* was isolated from a single broiler meat sample out of five collected from a local market. This isolate exhibited an alarming AMR profile, showing 100% resistance to almost all antibiotics tested—including ampicillin, penicillin g, streptomycin, vancomycin, cephotaxime, ciprofloxacin, erythromycin, bacitracin, and rifampicin. The only exception was chloramphenicol, to which it was fully susceptible. The calculated MAR index was 0.9 (52). In stark contrast, Natarajan et al. found *Klebsiella* in 9 out of 32 meat samples (30 from fresh meat and 2 from frozen products) (53). Interestingly, none of these isolates showed any resistance to the antibiotics tested. A more recent study by Vasanthi et al. adds nuance to these extremes. Out of 30 meat swab samples, 10 tested positive for *Klebsiella*. The AMR profile revealed moderate resistance: 33% of isolates were resistant to doxycycline, while 22% showed resistance to methicillin, penicillin, ampicillin, cefotaxime, and enrofloxacin. The lowest resistance was observed for ciprofloxacin (6%) (54).

***Aeromonas:*** *Aeromonas* species were identified in two additional studies from India, conducted in Madhya Pradesh (62) and the northeastern states of Meghalaya and Assam (63). In Madhya Pradesh, Kaskhedikar and Chhabra isolated *Aeromonas hydrophila* from retail chicken meat samples (62). The isolate displayed complete susceptibility (0% resistance) to a wide range of antibiotics, including ciprofloxacin, cefuroxime, ceftriaxone, cefotaxime, chloramphenicol, gentamicin, kanamycin, nitrofurantoin, nalidixic acid, and ofloxacin. However, significant resistance was observed for ampicillin (100%), colistin (100%), and oxytetracycline (50%). The calculated MAR index was 0.5 (62). In contrast, Sharma et al. reported *Aeromonas* isolates in 12 out of 104 meat samples from retail shops in Meghalaya and Assam. The isolates exhibited high resistance to several antibiotics: 100% resistance to ampicillin, kanamycin, chlortetracycline, tetracycline, and cephalothin; 91.67% resistance to sulphafurazole, carbenicillin, and trimethoprim; and notable resistance to co-trimoxazole (83.34%) and chloramphenicol (75%). On the other hand, lower resistance levels were observed for ciprofloxacin (8.34%), gentamicin (41.67%), and cephotaxime (25%). The MAR index was not reported (63).

***Proteus mirabillis:*** Two studies from southern India—Andhra Pradesh (64) and Tamil Nadu (52)—have reported the presence of *Proteus mirabilis* in chicken meat. Chinnam et al. identified *Proteus mirabilis* in 38 out of 195 meat samples collected in Andhra Pradesh, marking a prevalence rate of 19.48%. All isolates showed complete resistance (100%) to beta-lactam antibiotics. Molecular analysis confirmed the presence of multiple resistance genes, including *blaTEM*, *blaSHV*, *blaOXA*, *blaCIT*, and *blaFOX* (64). In Tamil Nadu, Modak et al. isolated *Proteus mirabilis* from 1 out of 5 broiler meat samples sold at a local market. This isolate demonstrated extensive resistance, with 100% resistance reported to most antibiotics tested, such as ampicillin, penicillin g, streptomycin, vancomycin, cephotaxime, erythromycin, bacitracin, rifampicin, and chloramphenicol. The only exception was ciprofloxacin, to which the isolate remained fully susceptible. The MAR index was calculated at 0.9 (52).

***Campylobacter:*** A recent study conducted by Suman et al. (13) in Karnataka assessed the prevalence and AMR profile of *Campylobacter* spp. in broiler chicken meat collected from slaughterhouses and retail meat shops. Out of 275 meat samples tested, 19 (6.9%) were positive for *Campylobacter*. The isolates exhibited high resistance to several antibiotics, with the highest rates observed for tetracycline (68.4%), doxycycline (63.2%), ampicillin (57.9%), and nalidixic acid (52.6%). Moderate resistance was found to kanamycin (36.8%), ciprofloxacin (31.6%), and co-amoxiclav (31.6%), while erythromycin had the lowest resistance at 26.3%. Resistance was linked to genetic mutations and the presence of specific resistance genes: mutations in codons 82, 161, and 120, along with A2074G and A2075G mutations in the *23S rRNA* gene. The *tetO* gene, responsible for tetracycline resistance, was found in 91.7% of resistant isolates, while the *blaOXA-61* gene, associated with ampicillin resistance, was detected in 59.2% of isolates—nearly all of which were phenotypically resistant (13).

***Enterobacter:*** In a 2022 study by Natarajan conducted in Tamil Nadu, *Enterobacter* species were isolated from chicken meat samples to assess their AMR profiles (53). A total of 32 samples (30 from fresh meat and 2 from frozen meat) were tested, out of which 5 isolates were identified as *Enterobacter* spp. Remarkably, all *Enterobacter* isolates exhibited zero resistance to the antibiotics tested (53).

***Pseudomonas:*** In the 2022 study by Natarajan from Tamil Nadu, *Pseudomonas* spp. was isolated from 9 out of 32 chicken meat samples (30 from fresh meat and 2 from frozen meat) (53). All *Pseudomonas* isolates exhibited 100% resistance to trimethoprim, sulfamethoxazole, and tigecycline. However, they were completely susceptible (0% resistance) to a wide range of critical and last-resort antibiotics, including amikacin, gentamicin (aminoglycosides), cefoperazone/Sulbactam, cefepime, ceftazidime (cephalosporins), imipenem, meropenem (carbapenems), minocycline, colistin, and piperacillin/tazobactam, ticarcillin/clavulanic acid (*β*-lactam/β-lactamase inhibitor combinations). The MAR index for *Pseudomonas* in this study was reported as 0.21 (53).

***Shigella:*** In a 2014 study conducted by Modak in Tamil Nadu, *Shigella* species were isolated from broiler chicken meat samples collected from local markets, with 2 out of 5 samples testing positive (52). The isolates displayed considerable resistance to a range of antibiotics. Notably, they exhibited 100% resistance to cephotaxime and bacitracin, while 50% resistance was observed against several other commonly used antibiotics including ampicillin, penicillin g, streptomycin, vancomycin, chloramphenicol, ciprofloxacin, erythromycin, and rifampicin. The MAR index was calculated as 1 (52).

### 3.2 Evidence of antibiotic-resistant gram-positive bacterial pathogens

*Staphylococcus aureus* emerged as the most frequently detected Gram-positive bacterium across the reviewed literature, being identified in 9 out of 32 studies (52, 53, 65–71), which represents 28.12% of all findings. While other Gram-positive organisms like *Micrococcus* spp. and *Listeria* spp. were occasionally isolated, they appeared infrequently. However, the geographical distribution of the studies that reported *S.aureus* was relatively narrow. Individual studies were conducted in Assam (65), Maharashtra (66), and Rajasthan (68), while Punjab (67, 70, 71) and Tamil Nadu (52, 53, 69) contributed three studies each. Particularly alarming data came from Tamil Nadu, where one study reported 100% resistance in *S aureus* isolates to a wide array of antibiotics, including commonly used drugs such as ampicillin, vancomycin, ciprofloxacin, and erythromycin (52). Another investigation in the same state observed resistance rates ranging from 7.5 to 100% (69), while additional research highlighted complete resistance to tetracycline and significant resistance (33.3%) to benzyl penicillin, clindamycin, and erythromycin, even in frozen meat (53).

Punjab contributed significantly to regional understanding, with three studies conducted between 2017 and 2024. In 2017, Herve analyzed 86 retail samples and isolated *S. aureus* in 46.51% of them. These isolates were completely resistant to cefpodoxime and cloxacillin, and showed high resistance to ceftazidime, piperacillin-tazobactam, methicillin, and clindamycin. Resistance to vancomycin and cefoxitin was also substantial (70%), although fluoroquinolones like ciprofloxacin and levofloxacin had lower resistance levels. Notably, linezolid remained effective in this study (67). By contrast, the 2024 study by Sharan reported a 92.3% isolation rate from 39 chicken meat samples. Alarmingly, all isolates were resistant to cefoxitin, confirming the presence of methicillin-resistant *S. aureus* (MRSA). Resistance to tetracycline, erythromycin, ampicillin, and even linezolid exceeded 80%. MDR was found in over 77% of isolates (70). A 2019 study by Zehra found lower levels of resistance, with penicillin (89.13%) and tetracycline (54.35%) being the most affected, and lower resistance rates for erythromycin (19.56%) and oxacillin (13.04%). Resistance to ceftriaxone and clindamycin was negligible, and no resistance was observed for vancomycin or chloramphenicol. The MDR rate was also comparatively lower (46%) (71).

In the northeastern state of Assam, Borah et al. examined 20 broiler chicken samples and found a high prevalence of *S. aureus* (70.59%), identifying 84 isolates. These isolates demonstrated complete resistance to several antibiotics, including methicillin, vancomycin, tetracycline, rifampicin, and erythromycin, indicating a widespread resistance pattern in that region (65). Similarly, in Maharashtra, Doiphode’s study recovered *S. aureus* from 36% of 50 retail chicken samples. The isolates were completely resistant to a broad range of antibiotics, covering multiple classes—beta-lactams, tetracyclines, macrolides, and lincosamides. Additionally, high resistance rates were noted for rifampicin, linezolid, vancomycin, and ciprofloxacin. Molecular analysis identified the presence of resistance genes such as *aacA-D*, *ermA*, and *tetK*/*tetM*, offering insight into the genetic mechanisms driving phenotypic resistance. The MAR index stood at a concerning 0.67 (66).

Finally, a 2015 study by Rao in Rajasthan reported *S. aureus* in 96% of 50 chicken samples. All isolates were resistant to ampicillin and cloxacillin, with high resistance levels also recorded for ofloxacin and tetracycline. Moderate resistance was observed for kanamycin and chloramphenicol, while erythromycin resistance was relatively low. Encouragingly, no resistance was seen to ciprofloxacin, doxycycline, and gentamicin (68).

***Micrococcus:*** In the study conducted by Modak et al. in Tamil Nadu, *Micrococcus* species was isolated from three out of five broiler chicken meat samples obtained from a local market (52). The AMR profile showed that these isolates exhibited complete resistance (100%) to streptomycin, vancomycin, erythromycin, and bacitracin. Partial resistance was observed against ampicillin (66.6%), penicillin g (33.3%), and rifampicin (33.3%). Notably, the isolates were fully susceptible (0% resistance) to cephotaxime, chloramphenicol, and ciprofloxacin. The calculated MAR index was 0.7 (52).

*Listeria*: In Beigh et al., conducted a study in Chhattisgarh, 100 meat samples collected from retail shops were tested for the presence of *Listeria* species (72). *Listeria* was isolated from 37% of the samples. All 37 isolates were tested for antimicrobial susceptibility, revealing a high prevalence of resistance. The isolates showed the highest resistance to norfloxacin (90.7%), followed by bacitracin, rifampicin, and colistin (each 81.4%). Moderate levels of resistance were noted for doxycycline hydrochloride (72%), erythromycin and gentamicin (69.8% each), penicillin-g and sulphadiazine (65% each), enrofloxacin (60.5%), and streptomycin (58%). Resistance was comparatively lower for ceftriaxone (46.5%), amoxicillin (18.6%), and chloramphenicol (11.6%). The MAR index was calculated at 0.82 (72).

## 4 Discussion

The rapid rise of antibiotic resistance around the world presents a serious challenge to the health of both humans and animals (73). This systematic review provides a comprehensive synthesis of data on the prevalence of bacterial pathogens in chicken meat samples across various Indian states, with a particular focus on their AMR patterns, pathogens and settings. The findings indicate that *E. coli* and *Salmonella* spp. are the most frequently reported gram-negative pathogens from retail chicken meat, with alarmingly high rates of resistance to commonly used antibiotics. Resistance to beta-lactams such as ampicillin and penicillin was widespread, and Tetracyclines and Fluoroquinolones also showed notably high resistance in several studies. Multiple studies reported MAR indices well above the threshold of 0.2, suggesting heavy exposure of these bacterial isolates to antimicrobial agents. The prevalence of MDR strains, including methicillin resistant *Staphylococcus aureus* (MRSA) and vancomycin-resistant *Staphylococcus aureus* (VRSA), further raises concerns regarding food safety and public health. Particularly resistance to beta-lactam antibiotics such as ampicillin and penicillin was consistently high across states.

Another notable finding of this review is the pronounced regional disparity in study representation and antimicrobial resistance profiles. For instance, studies from Tamil Nadu, Assam, and Maharashtra reported higher prevalence rates of *E.coli*, often coupled with MAR indices exceeding 0.9, indicating heavy antibiotic exposure. In contrast, studies from Bihar and West Bengal showed lower prevalence but still documented considerable resistance to older antibiotics like tetracyclines and chloramphenicol. However, this may result from less studies conducted in these states. Southern India, particularly Tamil Nadu, contributed the highest number of studies, reflecting both greater research activity and possibly higher awareness or surveillance infrastructure in this region. Interestingly, certain southern and northeastern states, such as Karnataka, Assam and Mizoram, though less represented, also reported the presence of methicillin resistant *Staphylococcus aureus* (MRSA) and extended-spectrum beta-lactamase (ESBL) -producing isolates, in their limited studies. This suggests that underrepresented regions might still be significant reservoirs of resistant bacteria. In contrast, several states such as Goa, Gujarat, Kerala, Odisha, and large parts of the northeastern and Himalayan belts lacked any published data, highlighting critical geographic gaps in national AMR monitoring efforts. Such uneven distribution may stem from disparities in public health infrastructure, academic research initiatives and funding, or poultry production intensity across different states of India.

The findings of our review resonate with global concerns surrounding AMR in foodborne pathogens, particularly those originating from poultry value chain and farming. Several studies conducted in this region have similar reports of *Escherichia coli* and *Salmonella* as dominant contaminants in chicken meat, often exhibiting multidrug resistance patterns (74–77). The high prevalence of *E.coli* across Indian states, coupled with resistance to first-line antibiotics like ampicillin, tetracycline, and streptomycin, mirrors trends observed in Bangladesh (78, 79), Vietnam (80, 81), and sub-Saharan Africa (82, 83), where overuse and misuse of antibiotics in poultry farming have been directly linked to resistant bacterial strains. Compared to high-income countries with stringent antibiotic use regulations and surveillance systems, India faces a compounded challenge due to its large poultry sector, weak enforcement of antibiotic stewardship, and over-the-counter access to veterinary antibiotics (84). The antibiotics with the highest resistance rates in our review- particularly beta-lactams (ampicillin/penicillin), tetracyclines, and older aminoglycosides/fluoroquinolones—are also among the most commonly used in Indian poultry production, both therapeutic and non-therapeutic use (85). Evidence from surveys and national reports indicates extensive on-farm use of tetracyclines and beta-lactams, as well as considerable use of fluoroquinolones (86). This widespread usage aligns with the high resistance rates observed in poultry meat isolates.

The widespread detection of MDR pathogens in Indian chicken meat poses significant public health risks, particularly for populations with high poultry consumption. The identification of high MAR indices in common zoonotic bacteria such as *E.coli*, *Salmonella*, *Staphylococcus aureus*, and *Campylobacter* is especially concerning, as these organisms are capable of causing not only gastrointestinal infections but also life-threatening systemic illnesses in humans (87). Resistance to critical classes of antibiotics—such as third-generation cephalosporins, fluoroquinolones, and even carbapenems in some isolates—threatens the effectiveness of lifesaving therapies and increases the risk of treatment failures (88, 89). Vulnerable populations, including the immunocompromised, the elderly, and children, face heightened exposure to these risks through contaminated meat. Additionally, the detection of AMR genes in bacteria isolated from both free-range and intensively farmed chickens illustrates that resistance is no longer limited to high-density farming environments (87).

Our current review highlights the urgent need for coordinated policy interventions and robust surveillance measures, anchored in a One Health framework (90). As a significant source of protein for millions of Indians, with weak regulatory enforcement around veterinary antibiotic use, the poultry industry becomes a critical focal point for national AMR containment efforts. The evidence from this review supports a multi-pronged One Health response that combines policy, surveillance, stewardship, farm-level biosecurity, and public engagement. Key actions include: (1) Strengthened surveillance — implement mandatory, harmonized AMR monitoring in poultry chains (farms, slaughterhouses, retail) using standardized sampling and antimicrobial susceptibility test protocols; results should be reported to a national AMR database to enable trend analysis and timely action. (2) Antimicrobial stewardship and regulation — restrict over-the-counter access to critically important antibiotics in veterinary practice, require prescription-only use, and phase out the use of medically important antimicrobials as growth promoters. (3) Veterinary diagnostics and farmer support — expand access to affordable, rapid diagnostic services and veterinary extension programs so treatments are targeted rather than empirical. (4) Farm-level measures — promote improved biosecurity, vaccination, husbandry practices, and waste management to reduce disease pressure and need for antibiotics; incentivize adoption via subsidies, certification or market premiums. (5) Capacity building and education — deliver training for veterinarians, para-veterinarians, and poultry producers on prudent antimicrobial use, record-keeping, and infection prevention. (6) Cross-sector coordination — establish regional One Health platforms linking public health, veterinary services, food safety authorities, agricultural extension, and environmental agencies with defined roles and data-sharing agreements. (7) Monitoring & evaluation — define key performance indicators (KPIs) such as (i) reduction in sales or defined daily doses (DDD) of priority antibiotics in poultry, (ii) prevalence of MDR isolates in sentinel sites, (iii) proportion of veterinary antibiotics dispensed by prescription, and (iv) number of farms adopting improved biosecurity. Implementation should follow a phased approach: starting with pilot surveillance in high-production states, parallel regulatory measures and stewardship training, then scaling nationally based on lessons learned. Strengthening the implementation of the National Action Plan on AMR (NAP-AMR) must include mandatory AMR surveillance in poultry meat, harmonized diagnostic protocols, and regular reporting systems (91). Geospatial analysis advocates for regionally tailored interventions in AMR hotspots, like high-risk states in India (92). Public education campaigns, responsible use of antibiotics in animal husbandry, and improved farm hygiene and waste management practices are also indispensable in curbing AMR transmission (93). Collectively these measures — implemented in an integrated One Health framework — will reduce selective pressure for resistance, improve the detection of emergent threats, and protect both animal and human health.

The WHO has introduced the AwaRe classification (Access, Watch, and Reserve) to guide the optimal use of antimicrobials and curb resistance development. In this framework, Access antibiotics (e.g., ampicillin, tetracyclines) are recommended as first-line treatments for common infections and should be widely available, whereas Watch antibiotics (e.g., fluoroquinolones and third-generation cephalosporins) are associated with higher resistance potential and should be used more judiciously. Reserve antibiotics (e.g., colistin, carbapenems) are considered last-resort options and should be preserved exclusively for the treatment of multi-resistant infections. The high resistance rates observed in our review to Access antibiotics such as ampicillin and tetracycline, and to Watch antibiotics such as ciprofloxacin, highlight an alarming trend that undermines WHO stewardship goals. Incorporating the AwaRe framework into national poultry health policies could help rationalize antibiotic use, promote stewardship, and protect the efficacy of last-resort antimicrobials.

### 4.1 Limitations

While this review offers a detailed synthesis of the prevalence and AMR patterns of bacterial pathogens in Indian chicken meat, several limitations must be acknowledged. The included studies exhibited considerable heterogeneity in methodologies, including variations in sample sizes, culture conditions, and antimicrobial susceptibility testing protocols, which limit direct comparison across studies. Additionally, geographic disparities in data availability were notable—certain states such as Tamil Nadu and Maharashtra were heavily represented, while many others, including Gujarat, Odisha, and much of the Northeast, lacked data altogether, reducing national representativeness. Furthermore, not all studies conducted molecular confirmation of resistance genes, thereby limiting insight into the genetic basis of AMR.

## 5 Conclusion

The findings of this review highlight a critical public health concern of AMR in India, where chicken meat is widely contaminated with MDR bacteria, including *E.coli, Salmonella, Klebsiella,* and *S. aureus*. The widespread nature of AMR in poultry pathogens underscores the urgent need for a coordinated One Health response that integrates surveillance, stewardship, and regulatory interventions across human, animal, and environmental sectors. In this context, aligning national antimicrobial usage policies with the WHO AwaRe classification is essential to ensure rational use of Access, Watch, and Reserve antibiotics, thereby preserving their efficacy. Continued research, improved data standardization, and expanded regional representation are necessary to fully address this growing threat and to safeguard both animal and human health.

## Footnotes

^1^ https://doi.org/10.17605/OSF.IO/MNQ9T
