# Supplementary material for: Prevalence, distribution and antimicrobial resistance profiles in poultry meat samples from India: a systematic review
Source: Front Vet Sci. 2025 Oct 31;12:1672628. doi: 10.3389/fvets.2025.1672628 (PMC12616860; doi:10.3389/fvets.2025.1672628)
Supplement: Supplementary file 1 [file Supplementary_file_1.docx]

Supplementary Material

Prevalence, distribution and antimicrobial resistance profiles in poultry meat samples from India: A Systematic review

Dr. Pooja Sajish V^1^, Dr. Nazim Uzzaman^1^, Niroshini Aramvalarthan^1^, Muhammad Asaduzzaman^2*^

^1^ Usher institute, University of Edinburgh, Edinburgh, UK

^2^ Department of Community Medicine and Global Health, Institute of Health and Society, Faculty of Medicine, University of Oslo, Norway

*** Correspondence:**Muhammad Asaduzzaman
[muhammad.asaduzzaman@medisin.uio.no](mailto:muhammad.asaduzzaman@medisin.uio.no)

Supplementary Table S1: PRISMA abstract checklist

Supplementary Table S2: Search strategy

Supplementary Table S3: Characteristics of primary studies

**Supplementary Table S1: PRISMA 2020 for Abstracts Checklist**(1)

| **Section and Topic** | **Item #** | **Checklist item** | **Reported (Yes/No)** |
| --- | --- | --- | --- |
| **TITLE** | | |  |
| Title | 1 | Identify the report as a systematic review. | Yes |
| **BACKGROUND** | | |  |
| Objectives | 2 | Provide an explicit statement of the main objective(s) or question(s) the review addresses. | Yes |
| **METHODS** | | |  |
| Eligibility criteria | 3 | Specify the inclusion and exclusion criteria for the review. | Yes |
| Information sources | 4 | Specify the information sources (e.g. databases, registers) used to identify studies and the date when each was last searched. | Yes |
| Risk of bias | 5 | Specify the methods used to assess risk of bias in the included studies. | No |
| Synthesis of results | 6 | Specify the methods used to present and synthesise results. | Yes |
| **RESULTS** | | |  |
| Included studies | 7 | Give the total number of included studies and participants and summarise relevant characteristics of studies. | Yes |
| Synthesis of results | 8 | Present results for main outcomes, preferably indicating the number of included studies and participants for each. If meta-analysis was done, report the summary estimate and confidence/credible interval. If comparing groups, indicate the direction of the effect (i.e. which group is favoured). | Yes |
| **DISCUSSION** | | |  |
| Limitations of evidence | 9 | Provide a brief summary of the limitations of the evidence included in the review (e.g. study risk of bias, inconsistency and imprecision). | Yes |
| Interpretation | 10 | Provide a general interpretation of the results and important implications. | Yes |
| **OTHER** | | |  |
| Funding | 11 | Specify the primary source of funding for the review. | No |
| Registration | 12 | Provide the register name and registration number. | No |

**Supplementary Table S2: Search Strategy**

Initial searches were conducted: Aug 12, 2024

| **PubMed** | **176** |
| --- | --- |
| ("drug resistance, microbial"[MeSH Terms] OR ((("antimicrobial"[Title/Abstract] OR "anti-microbial"[Title/Abstract]) AND ("resist*"[Title/Abstract] OR "susceptibility"[Title/Abstract])) OR (("antibiotic"[Title/Abstract] OR "anti-biotic"[Title/Abstract]) AND ("resist*"[Title/Abstract] OR "susceptibility"[Title/Abstract])) OR (("antibacterial"[Title/Abstract] OR "anti-bacterial"[Title/Abstract]) AND ("resist*"[Title/Abstract] OR "susceptibility"[Title/Abstract]))) OR ("multidrug"[Title/Abstract] AND "resistant*"[Title/Abstract]) OR "Antibiogram"[Title/Abstract] OR "resistant genes"[Title/Abstract])  AND  ("Foodborne Diseases"[MeSH Terms] OR ("Poultry"[MeSH Terms] OR "Poultry Products"[MeSH Terms] OR "Poultry"[Title/Abstract] OR "chicken"[Title/Abstract] OR "meat products"[Title/Abstract] OR "food born* pathogen*"[Title/Abstract]) OR "food safety"[Title/Abstract])  AND  ("India"[MeSH Terms] OR "India"[Title/Abstract] OR "Andhra Pradesh"[Title/Abstract] OR "Arunachal Pradesh"[Title/Abstract] OR "Assam"[Title/Abstract] OR "Bihar"[Title/Abstract] OR "Chhattisgarh"[Title/Abstract] OR "Goa"[Title/Abstract] OR "Gujarat"[Title/Abstract] OR "Haryana"[Title/Abstract] OR "Himachal Pradesh"[Title/Abstract] OR "Jharkhand"[Title/Abstract] OR "Karnataka"[Title/Abstract] OR "Kerala"[Title/Abstract] OR "Madhya Pradesh"[Title/Abstract] OR "Maharashtra"[Title/Abstract] OR "Manipur"[Title/Abstract] OR "Meghalaya"[Title/Abstract] OR "Mizoram"[Title/Abstract] OR "Nagaland"[Title/Abstract] OR "Odisha"[Title/Abstract] OR "Punjab"[Title/Abstract] OR "Rajasthan"[Title/Abstract] OR "Sikkim"[Title/Abstract] OR "Tamil Nadu"[Title/Abstract] OR "Telangana"[Title/Abstract] OR "Tripura"[Title/Abstract] OR "Uttarakhand"[Title/Abstract] OR "Uttar Pradesh"[Title/Abstract] OR "West Bengal"[Title/Abstract] OR "Hindustan"[Title/Abstract] OR "Bharat"[Title/Abstract]) |  |
| **Web of Science** | **766** |
| **(TS=("drug resistance, microbial" OR** **(("antimicrobial" OR "anti-microbial") AND ("resist*" OR "susceptibility")) OR** **(("antibiotic" OR "anti-biotic") AND ("resist*" OR "susceptibility")) OR** **(("antibacterial" OR "anti-bacterial") AND ("resist*" OR "susceptibility")) OR** **("multidrug" AND "resistant*") OR** **"Antibiogram" OR** **"resistant genes")) OR AB=(antimicrobial resistance)**  **AND**  **(TS=("Foodborne Diseases" OR** **"Poultry" OR** **"Poultry Products" OR** **"Poultry" OR** **"chicken" OR** **"meat products" OR** **"food born* pathogen*" OR** **"food safety"))**  **AND**  **(ALL=("India" OR** **"Andhra Pradesh" OR** **"Arunachal Pradesh" OR** **"Assam" OR** **"Bihar" OR** **"Chhattisgarh" OR** **"Goa" OR** **"Gujarat" OR** **"Haryana" OR** **"Himachal Pradesh" OR** **"Jharkhand" OR** **"Karnataka" OR** **"Kerala" OR** **"Madhya Pradesh" OR** **"Maharashtra" OR** **"Manipur" OR** **"Meghalaya" OR** **"Mizoram" OR** **"Nagaland" OR** **"Odisha" OR** **"Punjab" OR** **"Rajasthan" OR** **"Sikkim" OR** **"Tamil Nadu" OR** **"Telangana" OR** **"Tripura" OR** **"Uttarakhand" OR** **"Uttar Pradesh" OR** **"West Bengal" OR** **"Hindustan" OR** **"Bharat"))** |  |
| **Scopus** | **568** |
| ( TITLE-ABS ( "india" ) OR TITLE-ABS ( "arunachal pradesh" ) OR TITLE-ABS ( "andhra pradesh" ) OR TITLE-ABS ( "assam" ) OR TITLE-ABS ( "bihar" ) OR TITLE-ABS ( "chhattisgarh" ) OR TITLE-ABS ( "goa" ) OR TITLE-ABS ( "gujarat" ) OR TITLE-ABS ( "haryana" ) OR TITLE-ABS ( "himachal pradesh" ) OR TITLE-ABS ( "jharkhand" ) OR TITLE-ABS ( "karnataka" ) OR TITLE-ABS ( "kerala" ) OR TITLE-ABS ( "madhya pradesh" ) OR TITLE-ABS ( "maharashtra" ) OR TITLE-ABS ( "manipur" ) OR TITLE-ABS ( "meghalaya" ) OR TITLE-ABS ( "mizoram" ) OR TITLE-ABS ( "nagaland" ) OR TITLE-ABS ( "odisha" ) OR TITLE-ABS ( "punjab" ) OR TITLE-ABS ( "rajasthan" ) OR TITLE-ABS ( "sikkim" ) OR TITLE-ABS ( "tamil nadu" ) OR TITLE-ABS ( "telangana" ) OR TITLE-ABS ( "tripura" ) OR TITLE-ABS ( "uttarakhand" ) OR TITLE-ABS ( "uttar pradesh" ) OR TITLE-ABS ( "west bengal" ) OR TITLE-ABS ( "hindustan" ) OR TITLE-ABS ( "bharat" ) ) AND ( antimicrobial AND resist* OR TITLE-ABS ( antimicrobial ) OR TITLE-ABS ( "anti-microbial" ) OR TITLE-ABS ( "anti-biotic" ) OR TITLE-ABS ( antibiotic ) OR TITLE-ABS ( "anti-bacterial" ) OR TITLE-ABS ( antibacterial ) OR TITLE-ABS ( "multi-drug" ) OR TITLE-ABS ( antibiogram ) OR TITLE-ABS ( "resistant genes" ) OR TITLE-ABS ( resist* ) OR TITLE-ABS ( susceptibility ) OR TITLE-ABS ( resistant* ) OR TITLE-ABS ( "resistant gene" ) OR TITLE-ABS ( antibiogram ) ) AND ( "food-borne diseases" OR "foodborne diseases" OR TITLE-ABS ( poultry ) OR TITLE-ABS ( "poultry products" ) OR TITLE-ABS ( chicken ) OR TITLE-ABS ( broiler ) OR TITLE-ABS ( "meat products" ) OR TITLE-ABS ( "foodborne pathogen" ) OR TITLE-ABS ( "food-borne pathogen" ) OR TITLE-ABS-KEY ( "food safety" ) ) |  |
| **Google Scholar** | **737** |
| **Group 1** |  |
| **Search 1a**: With **all** of the words: “antimicrobial resist*”  Exact phrase: Poultry  With **at least one** of the words: India Andhra Pradesh Arunachal Pradesh Assam Bihar Chhattisgarh Goa Gujarat Haryana Himachal Pradesh Jharkhand Karnataka Kerala Madhya Pradesh Maharashtra |  |
| **Search 1b**: With **all** of the words: “antimicrobial resist*”  Exact phrase: Poultry  With **at least one** of the words: Manipur Meghalaya Mizoram Nagaland Odisha Punjab Rajasthan Sikkim Tamil Nadu Telangana Tripura Uttarakhand Uttar Pradesh West Bengal Hindustan Bharat |  |
| **Search 2a**: With **all** of the words: “antimicrobial resist*”  Exact phrase: chicken  With **at least one** of the words: India Andhra Pradesh Arunachal Pradesh Assam Bihar Chhattisgarh Goa Gujarat Haryana Himachal Pradesh Jharkhand Karnataka Kerala Madhya Pradesh Maharashtra |  |
| **Search 2b**: With **all** of the words: “antimicrobial resist*”  Exact phrase: chicken  With **at least one** of the words: Manipur Meghalaya Mizoram Nagaland Odisha Punjab Rajasthan Sikkim Tamil Nadu Telangana Tripura Uttarakhand Uttar Pradesh West Bengal Hindustan Bharat |  |
| **Search 3a**: With **all** of the words: “antimicrobial resist*”  Exact phrase: "food born* pathogen*"  With **at least one** of the words: India Andhra Pradesh Arunachal Pradesh Assam Bihar Chhattisgarh Goa Gujarat Haryana Himachal Pradesh Jharkhand Karnataka Kerala Madhya Pradesh Maharashtra |  |
| **Search 3b:** With **all** of the words: “antimicrobial resist*”  Exact phrase: "food born* pathogen*"  With **at least one** of the words: Manipur Meghalaya Mizoram Nagaland Odisha Punjab Rajasthan Sikkim Tamil Nadu Telangana Tripura Uttarakhand Uttar Pradesh West Bengal Hindustan Bharat |  |
| **Group 2** |  |
| **Search 1a:** With **all** of the words: “antimicrobial susceptibility”  Exact phrase: Poultry  With **at least one** of the words: India Andhra Pradesh Arunachal Pradesh Assam Bihar Chhattisgarh Goa Gujarat Haryana Himachal Pradesh Jharkhand Karnataka Kerala Madhya Pradesh Maharashtra |  |
| **Search 1b:** With **all** of the words: “antimicrobial susceptibility”  Exact phrase: Poultry  With **at least one** of the words: Manipur Meghalaya Mizoram Nagaland Odisha Punjab Rajasthan Sikkim Tamil Nadu Telangana Tripura Uttarakhand Uttar Pradesh West Bengal Hindustan Bharat |  |
| **Search 2a:** With **all** of the words: “antimicrobial susceptibility”  Exact phrase: chicken  With **at least one** of the words: India Andhra Pradesh Arunachal Pradesh Assam Bihar Chhattisgarh Goa Gujarat Haryana Himachal Pradesh Jharkhand Karnataka Kerala Madhya Pradesh Maharashtra |  |
| **Search 2b:** With **all** of the words: “antimicrobial susceptibility”  Exact phrase: chicken  With **at least one** of the words: Manipur Meghalaya Mizoram Nagaland Odisha Punjab Rajasthan Sikkim Tamil Nadu Telangana Tripura Uttarakhand Uttar Pradesh West Bengal Hindustan Bharat |  |
| **Search 3a:** With **all** of the words: “antimicrobial susceptibility”  Exact phrase: "food born* pathogen*"  With **at least one** of the words: India Andhra Pradesh Arunachal Pradesh Assam Bihar Chhattisgarh Goa Gujarat Haryana Himachal Pradesh Jharkhand Karnataka Kerala Madhya Pradesh Maharashtra |  |
| **Search 3b:** With **all** of the words: “antimicrobial susceptibility”  Exact phrase: "food born* pathogen*"  With **at least one** of the words: Manipur Meghalaya Mizoram Nagaland Odisha Punjab Rajasthan Sikkim Tamil Nadu Telangana Tripura Uttarakhand Uttar Pradesh West Bengal Hindustan Bharat |  |
| **Group 3** |  |
| **Search 1a:** With **all** of the words: “antibiotic susceptibility”  Exact phrase: Poultry  With **at least one** of the words: India Andhra Pradesh Arunachal Pradesh Assam Bihar Chhattisgarh Goa Gujarat Haryana Himachal Pradesh Jharkhand Karnataka Kerala Madhya Pradesh Maharashtra |  |
| **Search 1b:** With **all** of the words: “antibiotic susceptibility”  Exact phrase: Poultry  With **at least one** of the words: Manipur Meghalaya Mizoram Nagaland Odisha Punjab Rajasthan Sikkim Tamil Nadu Telangana Tripura Uttarakhand Uttar Pradesh West Bengal Hindustan Bharat |  |
| **Search 2a:** With **all** of the words: “antibiotic susceptibility”  Exact phrase: chicken  With **at least one** of the words: India Andhra Pradesh Arunachal Pradesh Assam Bihar Chhattisgarh Goa Gujarat Haryana Himachal Pradesh Jharkhand Karnataka Kerala Madhya Pradesh Maharashtra |  |
| **Search 2b:** With **all** of the words: “antibiotic susceptibility”  Exact phrase: chicken  With **at least one** of the words: Manipur Meghalaya Mizoram Nagaland Odisha Punjab Rajasthan Sikkim Tamil Nadu Telangana Tripura Uttarakhand Uttar Pradesh West Bengal Hindustan Bharat |  |
| **Search 3a:** With **all** of the words: “antibiotic susceptibility”  Exact phrase: "food born* pathogen*"  With **at least one** of the words: India Andhra Pradesh Arunachal Pradesh Assam Bihar Chhattisgarh Goa Gujarat Haryana Himachal Pradesh Jharkhand Karnataka Kerala Madhya Pradesh Maharashtra |  |
| **Search 3b:** With **all** of the words: “antibiotic susceptibility”  Exact phrase: "food born* pathogen*"  With **at least one** of the words: Manipur Meghalaya Mizoram Nagaland Odisha Punjab Rajasthan Sikkim Tamil Nadu Telangana Tripura Uttarakhand Uttar Pradesh West Bengal Hindustan Bharat |  |
| **Group 4** |  |
| **Search 1a:** With **all** of the words: “antibiogram”  Exact phrase: Poultry  With **at least one** of the words: India Andhra Pradesh Arunachal Pradesh Assam Bihar Chhattisgarh Goa Gujarat Haryana Himachal Pradesh Jharkhand Karnataka Kerala Madhya Pradesh Maharashtra |  |
| **Search 1b:** With **all** of the words: “antibiogram”  Exact phrase: Poultry  With **at least one** of the words: Manipur Meghalaya Mizoram Nagaland Odisha Punjab Rajasthan Sikkim Tamil Nadu Telangana Tripura Uttarakhand Uttar Pradesh West Bengal Hindustan Bharat |  |
| **Search 2a:** With **all** of the words: “antibiogram”  Exact phrase: chicken  With **at least one** of the words: India Andhra Pradesh Arunachal Pradesh Assam Bihar Chhattisgarh Goa Gujarat Haryana Himachal Pradesh Jharkhand Karnataka Kerala Madhya Pradesh Maharashtra |  |
| **Search 2b:** With **all** of the words: “antibiogram”  Exact phrase: chicken  With **at least one** of the words: Manipur Meghalaya Mizoram Nagaland Odisha Punjab Rajasthan Sikkim Tamil Nadu Telangana Tripura Uttarakhand Uttar Pradesh West Bengal Hindustan Bharat |  |
| **Search 3a:** With **all** of the words: “antibiogram”  Exact phrase: "food born* pathogen*"  With **at least one** of the words: India Andhra Pradesh Arunachal Pradesh Assam Bihar Chhattisgarh Goa Gujarat Haryana Himachal Pradesh Jharkhand Karnataka Kerala Madhya Pradesh Maharashtra |  |
| **Search 3b:** With **all** of the words: “antibiogram”  Exact phrase: "food born* pathogen*"  With **at least one** of the words: Manipur Meghalaya Mizoram Nagaland Odisha Punjab Rajasthan Sikkim Tamil Nadu Telangana Tripura Uttarakhand Uttar Pradesh West Bengal Hindustan Bharat |  |

**Supplementary Table S3: Characteristics of primary studies**

| **Study** | **Study area** | **Data collection time** | **Sample size** | **Pathogens studied** | **Type of bacteria** | **Study setting** | **Antibiotic susceptibility test** | **Antimicrobial agents** | **Number of bacterial isolate** | **MAR index/MDR (%)** | **Resistance genes** |
| --- | --- | --- | --- | --- | --- | --- | --- | --- | --- | --- | --- |
| Beigh, 2019(2) | Chhattisgarh | 2017 | 100 | *Listeria* | Gram positive | Retail shop | Disc diffusion method | Amoxycillin (30 µg), Bacitracin (10 units), Chloramphenicol (30 µg), Ciprofloxacin (5µg), Colistin (10 µg), Ceftriaxone (30 µg), Ceftriaxone + Tazobactum (30 µg/10), Doxycycline hydrochloride (30 µg), Enrofloxacin (10 µg), Erythromycin (15 µg), Gentamicin (10 µg), Penicillin-G (10 units), Rifampicin (5µg), Norfloxacin (10 µg), Streptomycin (10 µg), Sulphadiazine (100 µg) and Tetracycline (30 µg). | 37 | Most of the isolates | NR |
| Bhardwaj, 2022(3) | Delhi Haryana | 2020 | 30 | *Salmonella* | Gram negative | Local market | Disc diffusion method | Ampicillin (10 µg), Penicillin (10 µg), Ciprofloxacin (5 µg), Erythromycin (15 µg), Tetracycline (30 µg), Imipenem (10 µg), Kanamycin (30 µg), Nalidixic acid (30 µg), Norfloxacin (10 µg), and Trimethoprim (5µg) | 14 | 100% | NR |
| Bhave, 2019(4) | Maharashtra | 2016 | 54 | *E.coli* | Gram negative | Retail shop | Disc diffusion method | Amoxycillin/clavulanic acid (30 µg), Ampicillin (10 µg), Chloramphenicol (30 µg), Ciprofloxacin (5 µg), Colistin (10 µg), Gentamicin (10 µg), Nalidixic acid (30 µg), Neomycin (30 µg), Tetracycline (30µg), and Trimethoprim (5 µg) | 32 | 0.9 | *blaTEM*  *blaCTX-M*  *blaOXA*  *iucD*  *kpsMTII* |
| Borah, 2016(5) | Assam | 2014 | 20 | *Staphylococcus aureus* | Gram positive | Retail shop | Disc diffusion method | Methicillin (5 µg), Vancomycin (30 µg), Metropenem (10 µg), Polymixin B (100 units), Metronidazole (5 µg), Novobiocin (30 µg), Cefixime (5 µg), Ciprofloxacin (5 µg), Tetracycline (30 µg), Gentamycin (30 µg), Amikacin (30 µg), Rifampicin (5 µg), Erythromycin (15 µg) and Ampicillin (10 µg) | 34 | 70.59% | NR |
| Chakravarty, 2015(6) | Andhra Pradesh | 2013 | NR | *E.coli* | Gram negative | Slaughter house | Disc diffusion method | Ampicillin, Chloramphenicol, Ciprofloxacin, Cotrimoxazole, Gentamicin, Nalidixixc acid, Nitrofurantoin, Penicillin-G, Norflaxacin and Tetracycline | NR | NR | NR |
| Chinnam, 2021(7) | Andhra Pradesh | 2017-2019 | 195 | *Proteus mirabillis* | Gram negative | NR | Disc diffusion method | Cefotaxime (30 μg), Ceftazidime (30 μg), Ceftriaxone (30 μg), and Aztreonam (30 μg) | 38 | NR | *blaTEM blaSHV blaOXA blaCIT  blaFOX* |
| Debbarma, 2022(8) | Mizoram | 2020 | 90 | *E.coli* | Gram negative | Retail shop | Disc diffusion method | Ampicillin (10 µg), Amoxycillin (10 µg), Norfloxacin (10 µg), Amoxyclav (30 µg), Tetracycline (30 µg), Cefazolin (30 µg), Ceftriaxone (30 µg), Cefotaxime (30 µg), Ciprofloxacin (5 µg), Cotrimoxazole (25 µg), Gentamicin (10 µg) and Imipenem (10 µg). | 72 | 56.94% | NR |
| Deka, 2008(9) | Assam | 2006 | 100 | *E.coli Salmonella* | Gram negative | Retail shop | Disc diffusion method | Ampicillin (10 µg), Streptomycin (10 µg), Gentamicin (10 µg), Tetracycline (30 µg), Oxytetracycline (30 µg), Cephalexin (30 µg), Cephotaxime (30 µg), Furazolidone (50 µg), Nitrofurantoin (300 µg), Nalidixic acid (30 µg), Ciprofloxacin (10 µg), Norfloxacin (10 µg), Chloramphenicol (300 µg), Colistin (10 µg), and Co-trimoxazole (25 µg). | 54  8 | NR | NR |
| Deka, 2022(10) | Assam | 2020 | 110 | *E.coli* | Gram negative | Local market | Disc diffusion method | Ampicillin (10 µg), Imipenem (10 µg), Cefotaxime (30 µg), Cefpodoxime (10 µg), Ceftazidime (30 µg), Cefoxitin (30 µg), Chloramphenicol (30 µg), Amikacin (30 µg), Streptomycin (10 µg), Nalidixic acid (30 µg) and Tetracycline (30 µg) | 98 | 86% | *ESBL genes* |
| Doiphode, 2022(11) | Maharashtra | 2020 | 50 | *Staphylococcus aureus* | Gram positive | Retail shop | Disc diffusion method | Penicillin, Methicillin, Oxacillin, Amoxyclav, Ampicillin, tetracycline, Oxytetracycline, Doxyxyxline, Gentamicin, Amikacin, Tobramycin, Streptomycin, Erythromycin, Clindamycin, Vancomycin, Teicoplanin, Chloramphenicol, Ciprofloxacin, Rifampicin, Fusidic acid, Trimethoprim, Mupirocin, Linazolid, Fosfomycin | 18 | 100% | *aacA-D  tetM*  *ermA*  *tetK* |
| Herve, 2017(12) | Punjab | 2016 | 86 | *Staphylococcus aureus* | Gram positive | Retail shop | Disc diffusion method | Amikacin (30 µg), Amoxycillin/Clavulanic acid (30 µg), Cefpodoxime (10 µg), Ciprofloxacin (5 µg/disc), Cefoxitin (30 µg), Ceftazidime (30 µg), Doxycycline Hydrochloride (30 µg), Methicillin (5 µg), Levofloxacin (5 µg) , Ofloxacin (5 µg), Piperacillin/Tazobactam (10 µg), Vancomycin (30 µg), Meropenem (10 µg), Gentamicin (10 µg), (Clarithromycin (15 µg), Clindamycin (2 µg), Teicoplanin (30 µg), Azithromycin (30 µg), Linezolid (15 µg), Cefotaxime (30 µg), Coxacillin (1 µg) and Chloramphenicol (30 µg) | 40 | Few isolates | *NR* |
| Hussain, 2017(13) | Karnataka Telangana Andhra Pradesh Maharashtra | 2015 | 45 | *E.coli* | Gram negative | Retail shop | Disc diffusion method | Fosfomycin, Gentamicin, Ciprofloxacin, Co-trimoxazole, Tetracycline, and Chloramphenicol | 40 | 65% | *blaCTX-M-15  blaSHV  blaTEM* |
| Indrajith, 2015(14) | Tamil Nadu | 2013 | 18 | *Salmonella* | Gram negative | Retail shop | Disc diffusion method | Aztreonam (30 µg), Cefotaxime (30 µg), Rifampicin (5 µg), Tetracycline (30 µg), Vancomycin (30 µg), Ampicillin (10 µg), Nalidixic acid (30 µg), Ciprofloxacin (5 µg) and Trimethoprim (25 µg). | 90 | 63% | NR |
| Jana, 2013(15) | West Bengal | 2004 | 83 | *E.coli* | Gram negative | Retail shop | Disc diffusion method | Amikacin, Cefixime, Chloramphenicol, Ciprofloxacin, Erythromycin, Gentamicin, Kanamycin, Methicillin, Novobiocin, Oxytetracycline, Sulphafurazole, Vancomycin | 33 | NR | NR |
| Jhandai, 2022(16) | Haryana | 2018-2019 | 50 | *E.coli* | Gram negative | Local market | Disc diffusion method | Amoxicillin-clavulanic acid (30 μg), Penicillin (10 μg), Cefoperazone (75 μg), Cefpodoxime (10 μg), Cefotaxime (30 μg), Ceftazidime (30 μg), Ceftriaxone (30 μg), Erythromycin (15 μg), Amikacin (30 μg), Gentamicin (10 μg), Streptomycin (10 μg), Chloramphenicol (30 μg), Tetracycline (30 μg), Aztreonam (30 μg) and Imipenem (10 μg) | 63 | 98.41% | NR |
| Kalwaniya, 2020(17) | Rajasthan | 2018 | 30 | *E.coli* | Gram negative | Retail shop | Disc diffusion method | Amoxyclav, Ampicillin, Ceftriaxone, Chloramphenicol, Co-trimoxazole, Enrofloxacin, Erythromycin, Gentamicin, Nalidixic acid and Oxytetracycline | 17 | NR | NR |
| Kaskhedikar, 2009(18) | Madhya Pradesh | 2007 | 15 | *Aeromonas* | Gram negative | Retail shop | Disc diffusion method | Ciprofloxacin, Cefuroxime, Ceftriaxone, Cefotaxime, Chloramphenicol, Gentamicin, Kanamycin, Nitrofurantoin, Nalidixic acid, Ofloxacin, Oxytetracycline, Ampicillin, Colistin | NR | 50% | NR |
| Kaushik, 2018(19) | Bihar | 2010-2013 | 228 | *E.coli* | Gram negative | Retail shop | Disc diffusion method | Ampicillin (10 µg), Amikacin (30 µg), Cefuroxime (30 µg), Ceftriaxone (30 µg), Cephalothin (30 µg), Ciprofloxacin (5 µg), Co‑trimoxazole (25 µg), Gentamicin (10 µg), Ofloxacin (5 µg), Penicillin‑G (10 units), Tetracycline (30 µg), and Vancomycin (30 µg). | 62 | 100% | NR |
| Londhe, 2013(20) | Maharashtra | 2011 | 106 | *E.coli* | Gram negative | Retail shop | Disc diffusion method | Nalidixic acid, Tetracycline, Gentamicin, Cotrimoxazole, Chloramphenicol, Ciprofloxacin, and Cephotaxime | 45 | 86.66% | *tetA gene* |
| Maripandi, 2010(21) | Tamil Nadu | 2003-2005 | 578 | *Salmonella* | Gram negative | Retail shop | Disc diffusion method | Amikacin (30 µg), Ampicillin (10 µg) Erythromycin (15 µg), Cephalothin (30 µg), Tetracycline (30 µg), Cotrimoxazole (23.75 µg), Chloramphenicol (30 µg), Gentamycin (10 µg), Ciprofloxacin (5 µg), Amoxicillin (30µg), Kanamycin (30 µg) and Nalidixic acid (30 µg). | 92 | 100% | NR |
| Mishra, 2014(22) | Madhya Pradesh | 2012 | 60 | *Salmonella* | Gram negative | Retail shop | Disc diffusion method | Ciprofloxacin (10 µg), Ceftriaxone (10 µg), Neomycin (30 µg), Doxycycline (30 µg), Amoxycillin (10 µg), Furazolidone (50 µg), Cephalexin (20 µg), Oxytetracycline (30 µg), Chloramphenicol (30 µg), Amikacin (25 µg), Gentamycin (15 µg), Colistin (10 µg), Azithromycin (30 µg), Ceftiofur sodium (30 µg), Cotrimoxazole (25 µg), Tetracycline (25 µg), Chlorotetracycline (25 µg), Levofloxacin (10 µg), Enrofloxacin (10 µg) | 7 | NR | NR |
| Modak, 2014(23) | Tamil Nadu | 2012 | 35 | *Proteus mirabillis Micrococcus Salmonella E.coli Shigella Klebsiella Staphylococcus aureus* | Gram negative & Gram positive | Local market | Disc diffusion method | Ampicillin (10 µg /disc), Penicillin G, Streptomycin, Vancomycin, Cephotaxime, Chloramphenicol (30 mcg/disc), Ciprofloxacin (5 mcg/disc), Erythromycin, Bacitracin and Rifampicin (5 µg /disc) | 1 | 100% | NR |
| Naik, 2015(24) | Chhattisgarh | 2013-2014 | 200 | *Salmonella* | Gram negative | Retail shop | Disc diffusion method | Oxytetracycline (30 μg), Amoxycillin (10 μg), Cephalexin (30 μg), Ciprofloxacin (5 μg), Gentamicin (30 μg), Erythromycin (10 μg), Cefotaxime (10 μg), Nalidixic acid (30 μg), Ampicillin (10 μg), Ceftazidime (30 μg), Imipenem (10 μg), Amoxyclav (30 μg), Cefixime (5 μg), and Meropenem (10 μg) | 14 | NR | NR |
| Natarajan, 2022(25) | Tamil Nadu | 2020-2021 | 32 | *E.coli Klebsiella Salmonella Pseudomonas Enterobacter Staphylococcus aureus* | Gram negative & Gram positive | Retail shop | Disc diffusion method | Ampicillin, Benzylpenicillin, Oxacillin, Amikacin, Gentamicin, Cefoperazone/Sulbactam, Cefepime, Ceftazidime, Cefuroxime, Cefuroxime Axetil, Ceftriaxone, Tigecycline, Minocycline, Sulfonamide, Trimethoprim/Sulfamethoxazole, Nalidixic Acid, Levofloxacin, Ciprofloxacin, Sulfonamides, Nitrofurantoin, Piperacillin/Tazobactam, Ticarcillin/ Clavulanic Acid, Amoxicillin/Clavulanic Acid, Cefoxitin Screen, Linezolid, Daptomycin, Teicoplanin, Vancomycin, Rifampicin, Aztreonam, Imipenem, Meropenem, Ertapenem, Doripenem, Colistin, Erythromycin, Clindamycin, Inducible Clindamycin Resistance, Daptomycin, Vancomycin, Rifampicin, Linezolid | 50 | 33.3-50% | NR |
| Rajashekhara, 2017(26) | Karnataka | 2015 | 240 | *Salmonella* | Gram negative | Retail shop | Disc diffusion method | Amikacin, Cefotaxime, Ceftriaxone/tazobactum, Chloramphenicol, Ciprofloxacin, Enrofloxacin, Gentamicin, Cotrimaxazole, Polymixin-B, Nalidixic acid, Ampicillin, Ampicillin- Sulbactum, Colistin, Tetracycline, Cefadroxil, Streptomycin and Ceftazidime. | 13 | ? | NR |
| Rao, 2015(27) | Rajasthan | 2013 | 50 | *Staphylococcus aureus* | Gram positive | Retail shop | Disc diffusion method | Ampicillin, Chloramphenicol, Ciprofloxacin, Cloxacillin, Doxycycline, Erythromycin, Gentamicin, Kanamycin, Ofloxacin and Tetracycline | 48 | Most of the isolates | NR |
| Ruban, 2018(28) | Tamil Nadu | 2016 | 120 | *Staphylococcus Aureus* | Gram positive | Retail shop | Disc diffusion method | Methicillin, Oxacillin Cefoxitin, Vancomycin, Gentamicin, Neomycin, Kanamycin, Chloramphenicol, Co-trimoxazole, Ciprofloxacin, Polymyxin-B, Novobiocin, Erythromycin, Ampicillin, Amoxicillin, Tetracycline | 80 | 99.38% | *NR* |
| Sharan, 2024(29) | Punjab | 2022 | 39 | *Staphylococcus Aureus* | Gram positive | Repository of the Centre for One Health | Disc diffusion method | Ampicillin, Gentamicin, Cefoxitin, Chloramphenicol, Ciprofloxacin, Erythromycin, Linezolid, Sulpha-cotrimoxazole, and Tetracycline | 36 | 72.23% | NR |
| Sharma, 2009(30) | Meghalaya and Assam | 2007 | 104 | *Aeromonas* | Gram negative | Retail shop | Disc diffusion method | Ampicillin (10 μg), Kanamycin (10 μg), Sulphafurazole (300 μg), Amikacin (30 μg), Carbenicillin (100 μg), Streptomycin (25 μg),Chlortetracycline (30 μg), Trimethoprim (30 μg), Ciprofloxacin (5 μg), Tetracycline (10 μg), Gentamicin (30 μg), Co-trimoxazole (25 μg), Chloramphenicol (30 μg), Cephotaxime (30 μg), Cephalothin (30 μg), Cefuroxime (30 μg) and Nalidixic acid (30 μg) | 12 | NR | NR |
| Suman, 2023(31) | Karnataka | 2021 | 275 | *Campylobacter* | Gram negative | Slaughter Houses and Retail shop | Broth Microdilution Assay | Ampicillin, Co-amoxiclav, Ciprofloxacin, Doxycycline, Erythromycin, Kanamycin, Nalidixic acid and Tetracycline | 19 | 68.4% | *tetO*  *blaOXA-61 gene* |
| Vasanthi, 2023(32) | Tamil Nadu | 2022-2023 | 30 | *E.coli Klebsiella* | Gram negative | Local market | Disc diffusion method | Amoxiclav (amoxicillin/ clavulanic Acid), Ampicillin, Cefotaxime/ clavulanic acid, Ciprofloxacin, Doxycycline hydrochloride, Enrofloxacin, Gentamicin, Methicillin, Penicillin G, Streptomycin, Tetracycline | 20  10 | NR | NR |
| Zehra, 2019(33) | Punjab | 2017 | 147 | *Staphylococcus aureus* | Gram positive | Retail shop | Etest & Disc diffusion method | Oxacillin, Penicillin, Tetracycline, Chloramphenicol, Trimethoprim/sulfamethoxazole, Ceftriaxone, Gentamicin, Erythromycin, Ciprofloxacin, Vancomycin, Amoxyclav, Clindamycin | 46 | 45.65% | *mecA* |

**Note:** MAR: Multiple Antibiotic Resistance; MDR: Multi-drug resistance; NR: Not Reported

**REFERENCES:**

1. Page MJ, McKenzie JE, Bossuyt PM, Boutron I, Hoffmann TC, Mulrow CD, et al. The PRISMA 2020 statement: an updated guideline for reporting systematic reviews. BMJ. 2021;372:n71.

2. Beigh Q, Shakya S, Patyal A, Ali SL, Bhonsle D. Isolation, identification and antibiotic susceptibility profiling of Listeria spp. from raw chicken meat in durg district of Chhattisgarh, India. Journal of Animal Research. 2019;9(4):543-9.

3. Bhardwaj DK, Taneja NK, Taneja P, Patel P. Phenotypic and genotypic characterization of multi-drug resistant, biofilm forming, human invasive strain of<i> Salmonella</i> Typhimurium SMC25 isolated from poultry meat in India. Microbial Pathogenesis. 2022;173:12.

4. Bhave S, Kolhe R, Mahadevaswamy R, Bhong C, Jadhav S, Nalb, et al. Phylogrouping and antimicrobial resistance analysis of extraintestinal pathogenic <i>Escherichia coli</i> isolated from poultry species. Turkish Journal of Veterinary & Animal Sciences. 2019;43(1):117-26.

5. Borah D, Singh V, Gogoi B, Hazarika M, Rahman A. Prevalence of multidrug resistant (MDR) novel Enterococcus faecium strain VDR03 in broiler chicken meat samples collected from Dibrugarh Town, Assam (India). Research Journal of Microbiology. 2016;11(4):126-32.

6. Chakravarty MS, Ganesh P, Amaranth D, Shanthi Sudha B, Subhashini M. Escherichia coli-occurrence in the meat of shrimp, fish, chicken and mutton and its antibiotic resistance. European Journal of Experimental Biology. 2015;5(7):41-8.

7. Chinnam BK, Nelapati S, Tumati SR, Bobbadi S, Peddada VC, Bodempudi B. Detection of b-Lactamase-producing proteus mirabilis strains of animal origin in Andhra Pradesh, India and Their Genetic Diversity. Journal of Food Protection. 2021;84(8):1374-9.

8. Debbarma M, Deka D, ChaaTolenkhomba T, Rajesh JB. Microbiological Contamination of Retail Meat from Mizoram (India) with Special Reference to Molecular Detection and Multi-Drug Resistance of Escherichia coli. Indian Journal of Veterinary Sciences and Biotechnology. 2022;18(2):32-5.

9. Deka A, Barua A, Hazarika R, Khan M. Antibiogram of Escherichia coli and Salmonella isolates from chicken meat. 2008.

10. Deka J, Ahmed G, editors. Antimicrobial resistance in Escherichia coli isolates collected from poultry meat: An epidemiological surveillance study from Guwahati city2022 2022. RAYYAN-LABELS: Assam,E.coli,Poultry: Springer.

11. Doiphode A, Waskar V, Kolhe R, Kadam B, Mhase P. Prevalence, Virulence Traits and Antimicrobial Resistance Pattern of Staphylococcus aureus Isolated from Chicken. Indian Journal of Veterinary Sciences and Biotechnology. 2022;18(4):130-2.

12. Herve DT, Kumar G. Prevalence of Staphylococcus aureus in retail chicken meat samples in Jalandhar, Punjab. Research Journal of Pharmacy and Technology. 2017;10(1):281-5.

13. Hussain A, Shaik S, Ranjan A, anwar N, Tiwari SK, Majid M, et al. Risk of Transmission of Antimicrobial Resistant <i>Escherichia coli</i> from Commercial Broiler and Free-Range Retail Chicken in India. Frontiers in Microbiology. 2017;8:13.

14. Indrajith S, Athmanathan B, Subbaraj DK, Meganathan V, Arockiaraj JEE, Woodrowwilson JE, Karuppannan S. Incidence and antibiotic resistant profiles of pathogenic Salmonellaspp. from different environmental and food samples. Int J Microbiol Immunol Res. 2015;3(6):76-83.

15. Jana A, Mondal A. <i>Serotyping</i>, <i>pathogenicity and antibiogram of</i> Escherichia coli <i>isolated from raw poultry meat in West Bengal</i>, <i>India</i>. Veterinaria Italiana. 2013;49(4):361-5.

16. Jhandai P. Antimicrobial susceptibility testing of Escherichia coli isolated from chicken meat in Hisar, Haryana. 2022.

17. Kalwaniya MK. Prevalence and antibiogram of Escherichia coli isolated from meat and meat products. 2020.

18. Kaskhedikar M, Chhabra D. Multiple drug resistance of Aeromonas hydrophila isolates from chicken samples collected from Mhow and Indore city of Madhyapradesh. World. 2009;2(1):31-2.

19. Kaushik P, Kumari S, Dayal S, Kumar S. Antimicrobial resistance and molecular characterisation of E. coli from poultry in eastern India. Veterinaria Italiana. 2018;54(3):197-204.

20. Londhe S, Bhiogade P, Chaudhari B, Madale D, Patil M, Jagadale S, Bannalikar A. Plasmid profile and antimicrobial susceptibility of Escherichia coli isolates from poultry meat in Mumbai. 2013.

21. Marip, i A, Al-Salamah AA. Multiple-antibiotic resistance and plasmid profiles of Salmonella enteritidis isolated from retail chicken meats. American Journal of Food Technology. 2010;5(4):260-8.

22. Mishra P, Shukla S. Identification and sensitivity to antimicrobial agents of Salmonella isolated from poultry carcass. Int J Anim Vet Fish Allied Sci. 2014;1(1):34-40.

23. Modak A, Kumar V, Bhaskara Rao KV. Microbial quality assessment of broiler chicken meat and evaluation of antibiotic susceptibility profile of isolates from retail outlets of Vellore, Tamilnadu, India. Research Journal of Pharmaceutical, Biological and Chemical Sciences. 2014;5(5):1195-202.

24. Naik V, Shakya S, Patyal A, Gade N. Isolation and molecular characterization of Salmonella spp. from chevon and chicken meat collected from different districts of Chhattisgarh, India. Veterinary world. 2015;8(6):702.

25. Natarajan S, Nagarajan B, DineshKumar R, Ravich, ran M, Muniasamy P, et al. Occurrence Of Multi Drug Resistant Bacteria From Raw Chicken Meat Of South India Retail Markets. International Journal of Life Science and Pharma Research. 2022;12(1):L105-L11.

26. Rajashekhara DB, Fairoze MN, Karabasanavar N, Madhavaprasad CB, Shilpa AG, Kotresh AM, et al. Molecular detection and antibiogram of virulent <i>Salmonellae</i> isolated from chicken meat. Indian Journal of Animal Sciences. 2017;87(2):208-11.

27. Rao R, Joshi R, Maherch, ani S, Chaudhary AK, Kumar A, Kumari P. Enumeration and antibiotic resistance pattern of Staphylococcus aureus from raw chicken meat sold in Bikaner city. Journal of Pure and Applied Microbiology. 2015;9(2):1389-94.

28. Ruban SW, Babu RN, Abraham RJ, Senthilkumar T, Kumaraswamy P, Porteen K, Vemala G. Prevalence and Antimicrobial Susceptibility of Staphylococcus aureus Isolated from Retail Chicken Meat in Chennai, India. Journal of Animal Research. 2018;8(3):423-7.

29. Sharan M, Dhaka P, Bedi JS, Mehta N, Singh R. Assessment of biofilm-forming capacity and multidrug resistance in <i>Staphylococcus aureus</i> isolates from animal-source foods: implications for lactic acid bacteria intervention. Annals of Microbiology. 2024;74(1):14.

30. Sharma I, Kumar A, Pramanik AK. Isolation and identification of mesophillic aeromonas bacteria from meat and fish foods of north east India. Journal of Pure and Applied Microbiology. 2009;3(2):517-26.

31. Suman Kumar M, Ramees TP, Dhanze H, Gupta S, Dubal ZB, Kumar A. Occurrence and antimicrobial resistance of Campylobacter isolates from broiler chicken and slaughter house environment in India. Animal Biotechnology. 2023;34(2):199-207.

32. Vasanthi B, Parthiban M, Sarathch, ra G, Narendra Babu R, Brindha K. Molecular Characterization and Antimicrobial Resistance Profiling of Food Borne Pathogens from Chicken Meat in Tamil Nadu, India. Indian Veterinary Journal. 2023;100(10):35-8.

33. Zehra A, Gulzar M, Sing R, Kaur S, Gill JPS. Prevalence, multidrug resistance and molecular typing of methicillin-resistant <i>Staphylococcus aureus</i> (MRSA) in retail meat from Punjab, India. Journal of Global Antimicrobial Resistance. 2019;16:152-8.
